# Imaging of hypochlorous acid in mitochondria using an asymmetric near-infrared fluorescent probe with large Stokes shift†

**DOI:** 10.1039/d2sc03833a

**Published:** 2022-09-08

**Authors:** Wei Hu, Taotao Qiang, Chenchen Li, Longfang Ren, Fei Cheng, Baoshuai Wang, Mingli Li, Xinjian Song, Tony D. James

**Affiliations:** College of Bioresources and Materials Engineering, Shaanxi Collaborative Innovation Center of Industrial Auxiliary Chemistry & Technology, Shaanxi University of Science & Technology Xi'an 710021 China qiangtt515@163.com; Hubei Key Laboratory of Biological Resources Protection and Utilization, Hubei University for Nationalities Enshi 445000 China whxjsong@163.com; Department of Chemistry, University of Bath Bath BA27AY UK t.d.james@bath.ac.uk; School of Chemistry and Chemical Engineering, Henan Normal University Xinxiang 453007 China

## Abstract

Small-molecule near-infrared (NIR) imaging facilitates deep tissue penetration, low autofluorescence, non-invasive visualization, and a relatively simple operation. As such it has emerged as a popular technique for tracking biological species and events. However, the small Stokes shift of most NIR dyes often results in a low signal-to-noise ratio and self-quenching due to crosstalk between the excitation and emission spectra. With this research, we developed a NIR-based fluorescent probe WD-HOCl for hypochlorous acid (HOCl) detection using the NIR dye TJ730 as the fluorophore, which exhibits a large Stokes shift of 156 nm, with no crosstalk between the excitation and emission spectra. It contains acyl hydrazide as the responsive group and a pyridinium cation as the mitochondria-targeting group. The fluorescence intensity of WD-HOCl was enhanced by 30.1-fold after reacting with HOCl. Imaging studies performed using BV-2 cells indicated that WD-HOCl could be used for endogenous HOCl detection and imaging in living cells exposed to glucose and oxygen deprivation/reperfusion. Finally, we demonstrated that inhibiting the expression of NOX2 reduced the HOCl levels and the severity of oxidative stress during stroke in a mouse model.

## Abstract 2

Small-molecule near-infrared (NIR) imaging facilitates deep tissue penetration, low autofluorescence, non-invasive visualization, and a relatively simple operation.

## Graphical abstract

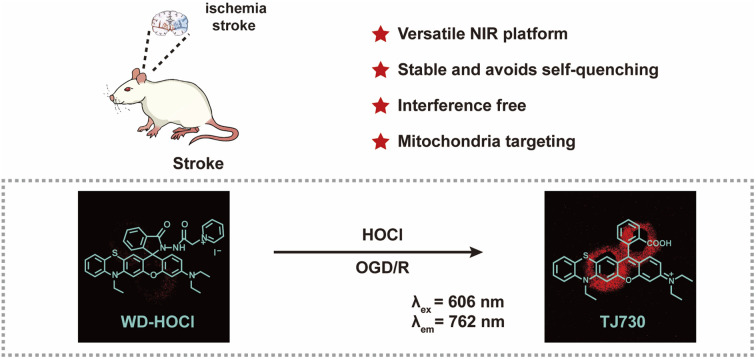

## Introduction

Near-infrared small-molecule fluorescent probes have been extensively developed due to their enhanced properties including deep tissue penetration, low autofluorescence, non-invasive visualization, and relatively simple operation,^1–3^ and as such have emerged as a popular technique for the real-time sensing and tracking of biological species and events.^4,5^ However, to improve their internal conversion efficiency, the HOMO–LUMO band gap of NIR dyes must be reduced. This task is difficult and often decreases the Stokes shift.^6^ Efforts to extend the Stokes shift include nitrogen insertion *via* cyanine or pyronine groups, twisted intramolecular charge transfer, and solvent cage relaxation. Unfortunately, none of these techniques can eliminate crosstalk between the excitation and emission spectra.^7–9^ Inducing a pseudo-Stokes shift based on energy transfer is complicated, is limited by the energy transfer efficiency, and dual emission peaks in the NIR region are difficult to achieve.^10,11^ These issues result in many disadvantages such as a low signal-to-noise ratio and self-quenching, making it difficult to increase the sensitivity and accuracy of imaging techniques.^12–14^ In contrast, the group of Yuan^15^ introduced asymmetric electronic structures to fluorescein, squaraine, cyanine, rhodamine, and oxazine dyes to improve their photophysical properties. Which was achieved by enhancing the internal conversion, reducing emission energy, and increasing the Stokes shift of fluorophores using vibronic contributions to the lowest unoccupied molecular orbital (LUMO). Inspired by this principle, we designed and synthesized a NIR dye containing a strong electron-donating heteroatom which increased the asymmetry of the electronic structure through vibronic contributions. Therefore, the NIR dye, TJ730, was used to prepare a novel NIR probe which eliminated crosstalk between excitation and emission spectra and improved the sensitivity and accuracy of imaging analysis.

Specifically, a novel heteroatom-substituted rhodamine-based NIR fluorescent probe for HOCl, named WD-HOCl, was developed. The probe consists of a fluorophore TJ730 decorated with acyl hydrazide as HOCl recognition group.^16^TJ730 exhibited a large Stokes shift (156 nm in acetonitrile) and achieved almost complete separation of its excitation and emission peaks. The fluorescence intensity of the NIR fluorescent probe was enhanced 30.1-fold upon reacting with HOCl. The detection limit of the NIR probe towards HOCl was 1.3 nM. The NIR probe also exhibited high selectivity, good chemical stability, photostability, and low cytotoxicity. Furthermore, a pyridinium cation was introduced to the fluorescent system as a mitochondria-targeting group. Thus, WD-HOCl can specifically localize in mitochondria to image endogenous HOCl and can sensitively and accurately track cerebral ischemia-reperfusion injury (CIRI)-induced oxidative stress processes.

## Results and discussion

### Design and synthesis of WD-HOCl

**Fig. 1:**
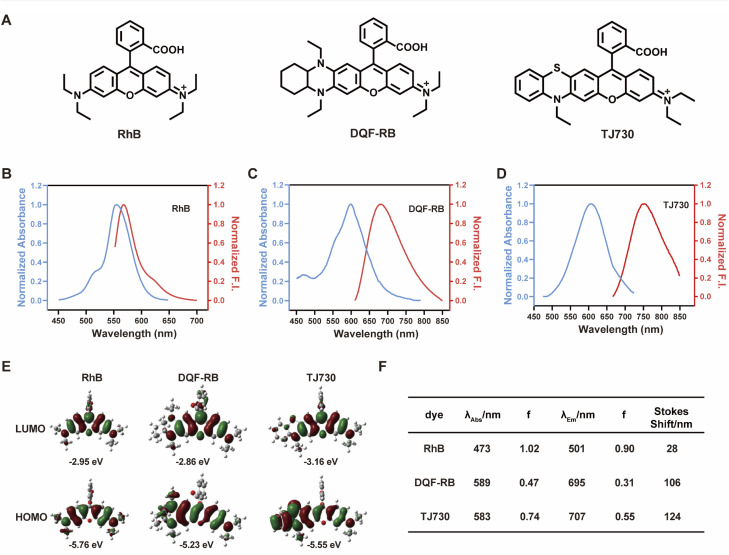
(A) Chemical structures and normalized absorption (black line)/emission (red line) spectra of RhB (B), DQF-RB (C), and TJ730 (D) in acetonitrile. (E) Density functional theory-optimized molecular orbital plots (lowest occupied molecular orbital and highest occupied molecular orbital) of RhB, DQF-RB, and TJ730. (F) Calculated absorption and emission wavelengths and oscillator strengths of the dyes (PCM solvation model with acetonitrile as the solvent).

Our concept was to increase the Stokes shift and thus minimize the overlap between the excitation and emission signals by introducing a S atom as an electron-donating group. Furthermore, the π-conjugation of the xanthene moiety was extended by incorporating aromatic rings to red-shift the excitation/emission wavelengths of the fluorophore.^17^ The fluorescent characteristics of the fluorophore TJ730 and the rhodamine-like fluorophores RhB and DQF-RB in acetonitrile solutions were investigated. As presented in Table 1 and Fig. 1A, RhB exhibited a maximum absorption peak at 553 nm and a corresponding emission maximum centered at 567 nm. Thus, its Stokes shift was only 14 nm, and as such extensive absorption and emission overlap was observed (as shown in Fig. 1B and Table 1). DQF-RB exhibited one distinct absorption band centered at 597 nm (*ε* = 127 000 M^−1^ cm^−1^) and a corresponding emission maximum at 682 nm. Thus, the Stokes shift (85 nm) was substantially higher than that of RhB. However, considerable overlap was observed between the absorption and emission spectra (as shown in Fig. 1C and Table 1). Significantly, the retrofitted NIR dye TJ730 exhibited a maximum absorption peak at 606 nm and a maximum emission peak at 762 nm, providing a large Stokes shift of 156 nm (as shown in Fig. 1D and Table 1). The near-complete separation of the excitation and emission peaks can provide a higher signal-to-background ratio and minimize self-quenching during bioimaging. To gain deeper insight into the red-shifted emission and large Stokes shift of TJ730, its optimized geometries and electronic structures in the highest occupied molecular orbital (HOMO) and lowest unoccupied molecular orbital (LUMO) in acetonitrile solution were calculated using density functional theory (DFT) and time-dependent DFT at the B3LYP/6-31G(d) level. RhB displayed symmetrical HOMO and LUMO structures (Fig. 1E) and a high oscillation strength, but its Stokes shift was only 28 nm (Fig. 1F). However, when an *N*,*N*-diethylamino group was substituted with a 1,4-diethyl-decahydroquinoxaline (DQF-RB) or 4-ethyl-3,4-dihydro-2*H*-benzo[*b*][1,4]thiazine (TJ730) group, the calculated Stokes shifts increased to 106 and 124 nm, respectively (Fig. 1E). Comparing the molecular orbital plots indicates that although the LUMO of DQF-RB and TJ730 remained symmetrical, the HOMO displayed an asymmetrical structure, which increased the HOMO energy level compared with the initial symmetrical structure. Thus, electrons in the excited state reverted to a higher-energy vibrational state, resulting in a lower emission energy and larger Stokes shift. All calculated absorption and emission wavelengths were consistent with their corresponding experimental values (Fig. 1F), confirming the reliability of the computational results.

**Table 1:** Photophysical properties of RhB, DQF-RB, TJ730 in acetonitrile

Dye	*λ*_abs_ a (nm)	*λ*_em_ b (nm)	*ε* c (M^−1^ cm^−1^)	*Φ* d	Stokes shift (nm)	*ε* × *Φ*^e^ (M^−1^ cm^−1^)
RhB	553	567	109 100	0.7	14	76 370
DQF-RB	597	682	127 000	0.358	85	45 466
TJ730	606	762	628 000	0.047	156	29 516

a Maximum absorption wavelength.

b Maximum fluorescence emission wavelength.

c Molar absorptivity.

d Quantum yield.

e Brightness.

Next, we evaluated the chemical stability of TJ730 to ensure its suitability for the high-fidelity imaging of hypochlorous acid (HOCl). Several previous studies have demonstrated that S, as the most important component of mercaptan derivatives, can maintain the redox balance of cells by reducing oxidizing species.^18^ The methyl thioether group has frequently been used as a modulator of the redox cycle between HOCl and GSH.^19^ Therefore, we suspected that TJ730 could be easily destroyed by the endogenous ROS produced, leading to poor reliability and stability. As illustrated in Fig. S10A and B,† we evaluated the stability of TJ730 against various oxidants and nucleophiles including H_2_O_2_, O_2_˙^−^, H_2_S, HSO_3_^−^, NO, ONOO^−^, HOCl, and ˙OH. The results indicated that TJ730 had good stability and is suitable for HOCl detection. We hypothesize that the extended π-conjugated system decreased the activity of S and increased the chemical stability of the dye.

### Fluorometric detection of HOCl using WD-HOCl

The photophysical properties of TJ730 in 10 mM PBS and organic solvents with different polarities were investigated to optimize the detection system. Fig. S10C and D† shows the absorption and fluorescence spectra of TJ730, and the corresponding photophysical data are presented in Table S1.†TJ730 exhibits an absorption band between 606 nm and 615 nm, which was attributed to the π–π* transition of the ring-opened rhodamine conjugate. Upon excitation at 606 nm, TJ730 presented a characteristic NIR fluorescence emission *λ*_em_ ≥ 745 nm. The absorption and fluorescence spectra revealed a weak bathochromic shift upon increasing solvent polarity due to the high polarity of the molecule resulting from its D–π–A structure. The results also illustrated that TJ730 exhibited the best photophysical properties in acetonitrile, with an absorption maximum at 606 nm (*ε* = 0.35 × 10^4^ M^−1^ cm^−1^) and an emission maximum at 762 nm (*Φ* = 4.7%). The Stokes shift between the excitation and emission peaks exceeded 156 nm. We then decorated TJ730 with acyl hydrazide (which can be oxidized and hydrolyzed specifically by HOCl) and a pyridinium cation (which can anchor in the cell mitochondria due to the positive charge). Therefore, we obtained a novel heteroatom-substituted rhodamine-based NIR fluorescent probe for HOCl which we called WD-HOCl. TJ730 and WD-HOCl were readily synthesized, and their synthetic routes and characterization details are given in Scheme S1 and Fig. S1–S9.†

**Fig. 2:**
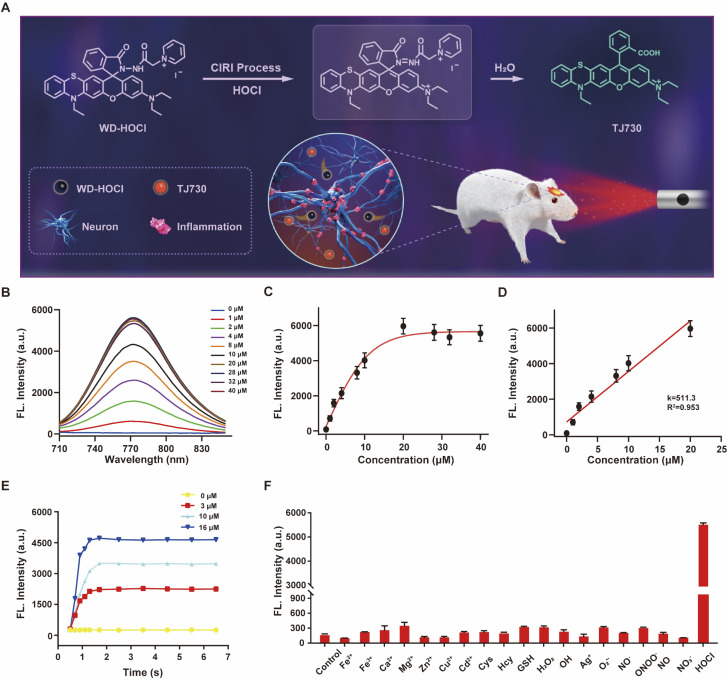
(A) Recognition mechanism of WD-HOCl. (B) Fluorescence spectra for WD-HOCl upon exposure to different amounts of HOCl (0–40 μM). (C) Fluorescence intensity of WD-HOCl at 762 nm as a function of the HOCl concentration. (D) Linear fluorescence response of WD-HOCl toward HOCl (0–20 μM). (E) Time-dependent reaction profiles of 10 μM WD-HOCl and (0, 3, 10, and 16 μM) HOCl. (F) Fluorescence responses of WD-HOCl to a blank control (a), 20 μM metal ions ((b–i) Fe^2+^, Fe^3+^, Ca^2+^, Mg^2+^, Zn^2+^, Ag^+^, Cu^2+^, and Cd^2+^, respectively), 1.0 mM biomolecules ((j–l) Cys, Hcy, and GSH, respectively), 100 μM ROS ((m–o), H_2_O_2_, ˙OH, and O_2_˙^−^, respectively), 100 μM reactive nitrogen species (RNS, (p–s) NO_2_^−^, ONOO^−^, NO, and NO_3_^−^, respectively), and 20 μM HOCl.

The proposed recognition and imaging mechanism of HOCl in mitochondria is given in Fig. 2A. WD-HOCl was sufficiently water-soluble for solution-phase detection and cell staining (Fig. S11†). The absorption and fluorescence spectra of WD-HOCl with and without HOCl in 10 mM PBS containing 33% acetonitrile were recorded. WD-HOCl displayed a negligible absorption peak at 596 nm owing to the closed-ring form of the probe. However, upon adding HOCl (2 eq.), a new absorption maximum appeared at 606 nm (*ε* = 1387 M^−1^ cm^−1^) (Fig. S12A†), indicating the ring-opening of TJ730. WD-HOCl is non-fluorescent however a strong emission peak at approximately 762 nm appeared immediately upon the addition of HOCl (2 eq.) (Fig. S12B†), indicating a large Stokes shift of about 156 nm. WD-HOCl was then used to quantify HOCl concentrations *in vitro*. Upon the addition of 2 eq. HOCl, a 30.1-fold enhancement of the fluorescence intensity was observed (Fig. 2B and C). Furthermore, a linear correlation between the fluorescence intensity of WD-HOCl at 762 nm and HOCl concentrations was observed (Fig. 2D). The detection limit of WD-HOCl for HOCl was calculated to be 1.3 nM, demonstrating its high sensitivity for the determination of HOCl. As presented in Fig. 2E, the reaction of WD-HOCl with HOCl (3, 10, and 16 μM) was nearly complete at 100 s, confirming that WD-HOCl exhibited a rapid response to HOCl. The mechanism for the interaction of WD-HOCl with HOCl was confirmed using high-resolution mass spectrometry (HRMS). The peak at *m*/*z* = 521.1863 was ascribed to the characteristic peak of the reaction product TJ730, and the peak at *m*/*z* = 654.2538 (representing the reactant) disappeared (Fig. S13†).

Selectivity is a critical factor for improving the performance of fluorescent probes for bioimaging applications. Thus, we investigated the fluorescence responses of WD-HOCl towards HOCl and 20 μM of potential interfering metal ions (Fe^2+^, Fe^3+^, Ca^2+^, Mg^2+^, Zn^2+^, Ag^+^, Cu^2+^, and Cd^2+^), 1.0 mM biomolecules (Cys, Hcy, and GSH), 100 μM ROS (H_2_O_2_, ˙OH, and O_2_˙^−^, respectively), 100 μM reactive nitrogen species (RNS, NO_2_^−^, ONOO^−^, NO, and NO_3_^−^). As illustrated in Fig. 2F, only negligible enhancements in the fluorescence intensity was caused by non-target species. However, the addition of HOCl remarkably enhanced the fluorescence, indicating that WD-HOCl was selective towards HOCl over other interferents. In addition, the fluorescence response of WD-HOCl towards HOCl was evaluated at different pH values (Fig. S14†). In the absence of HOCl, WD-HOCl exhibited no obvious fluorescence intensity changes over the pH range of 4.2–8.0, however, the fluorescence intensity fluctuates slightly after reaction with HOCl.

### Bioimaging applications of WD-HOCl in living cells

Potential applications of WD-HOCl for monitoring fluctuations in HOCl levels in living cells were then evaluated. Using a 3-(4,5-dimethylthiazol-2-yl)-2,5-diphenyltetrazolium bromide (MTT) assay, the cytotoxicity of WD-HOCl towards BV-2 cells was examined after being incubated with different concentrations (0, 2, 5, 10, and 15 μM) of WD-HOCl for 24 h. The resultant cell viabilities all exceeded 90%, confirming that WD-HOCl exhibited negligible cytotoxicity, even at a concentration of 10 μM (Fig. S15†). Furthermore, the photostability of WD-HOCl during the imaging of BV-2 cells was also investigated. The results indicate that the fluorescence intensity of WD-HOCl remained nearly constant for 20 min, confirming its photostability (Fig. S16†).

In order to more accurately reflect the fluctuation of HOCl in pathological process, it is necessary for the probe to anchor at the cell mitochondria. As an organelle with a double-membrane structure, the negative charge of the inner mitochondrial membrane enables positively charged molecules to be specifically enriched and anchored to the mitochondria. Since the pyridinium cation group of WD-HOCl has a positive charge, it should exhibit mitochondrial localization ability. To this end, WD-HOCl was mixed with a commercial mitochondrial localization dye, 100 nM Mito-Tracker Green, a commercial lysosome localization dye, 100 nM Lyso-Tracker Green, and a commercial endoplasmic reticulum localization dye, 100 nM ER-Tracker Green. These were co-incubated with BV-2 cells for 30 min and then imaged using a confocal microscope. The results in Fig. S17† indicate that WD-HOCl was mainly distributed in the mitochondria (the Pearson colocalization coefficient reached 0.94). The Pearson colocalization coefficients in the lysosome and endoplasmic reticulum were 0.31 and 0.33, respectively, indicating that WD-HOCl has a good mitochondrial localization ability, providing a good basis for studying ROS produced during respiration. To explore the biological applications of WD-HOCl, exogenous HOCl in BV-2 cells was determined. BV-2 cells were incubated with WD-HOCl (5 μM) for 30 min, washed with PBS, treated with different concentrations of NaOCl (0, 5, 10, and 20 μM) for another 30 min, and immediately imaged using a confocal fluorescence microscope (Fig. S18†). The fluorescence intensity in the red channel (*I*_red_) increased in a concentration-dependent manner, indicating that WD-HOCl was membrane-permeable and could be used to detect exogenous HOCl in living cells.

To detect endogenous HOCl using WD-HOCl, BV-2 cells were preconditioned with the ROS stimulant phorbol 12-myristate-13-acetate (PMA, 2 μg mL^−1^) for 1 h and then treated with WD-HOCl for another 30 min. As illustrated in Fig. S19,†*I*_red_ of the PMA-stimulated cells was substantially higher than that of cells treated with only the probe. Furthermore, *I*_red_ for the cells pretreated with HOCl scavengers including lipoic acid (5 μg mL^−1^), *N*-acetyl-l-cysteine (NAC, 2 μg mL^−1^) and l-methionine (1 μg mL^−1^) exhibit weak fluorescence. The experimental results confirm that WD-HOCl can be used to monitor endogenous HOCl in living cells.

**Fig. 3:**
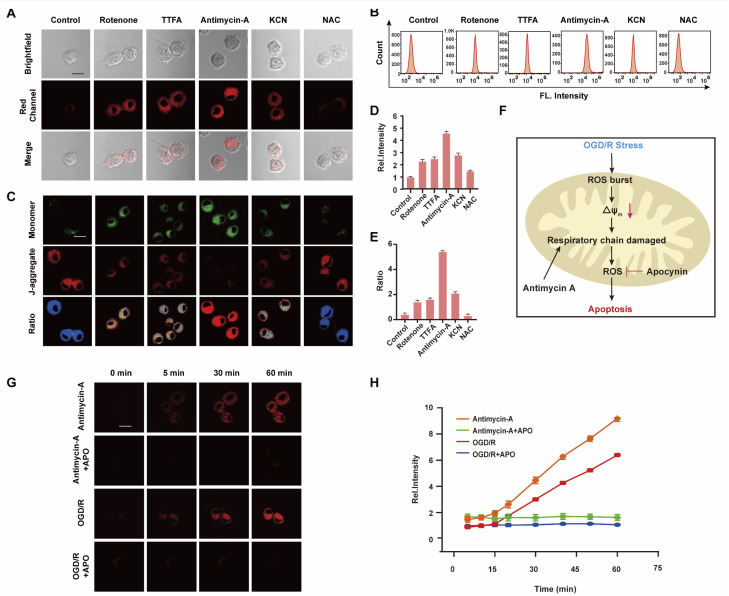
(A) Images of WD-HOCl (10 μM) loaded BV-2 cells with the mitochondrial respiratory inhibitor including control, 5 μM of rotenone, 5 μM of TTFA, 5 μM of antimycin A and 500 μM of malonic for 30 min. (B) Flow cytometry analysis of (A). (C) Images of mitochondrial membrane potential analyzed by JC-1. (D) Relative fluorescence intensities of BV-2 cells as used in (A). (E) Ratio intensities of BV-2 cells as used in (C). (F) Scheme of OGD/R-induced signal transduction pathways. Imaging (G) and time course of fluorescence ratio changes (H) of BV2 cells. Cells incubated with 10 μM WD-HOCl with the first line: antimycin A (5 μM); the second line: antimycin A + APO (10 mM); the third line: OGD/R and the fourth line: OGD/R + APO. For the fluorescent images, the experiment was repeated using three cultures; similar results were obtained each time. Scale bar = 20 μm. Emissions were collected at the red channel (680–738 nm) with 606 nm excitation. The error bars mean SD (*n* = 33 cells).

The mitochondrial respiratory chain is the chief source of HOCl generation.^20^ Complex I (NADH:ubiquinone oxidoreductase), complex II (succinate dehydrogenase), complex III (ubiquinol–cytochrome c reductase) and complex IV (cytochrome c oxidase) are all redox centers for HOCl generation. Mitochondrial respiratory inhibitors, such as rotenone (a complex I inhibitor), thenoyltrifluoroacetone (TTFA, a complex II inhibitor), antimycin A (a complex III inhibitor) and KCN (a complex IV inhibitor), were employed to block the respiratory chain selectively and induce mitochondrial HOCl overproduction. Remarkable *I*_red_ enhancement were observed for the four inhibitors *versus* that in the control group (Fig. 3A and D). In addition, the cells were incubated for 30 min with NAC to eliminate HOCl. The weak fluorescence in Fig. 3A and D illustrates a very low endogenous HOCl concentration in BV-2 cells. Flow cytometry analysis also indicated fluorescence intensity variations. Compared with the control group, significant fluorescence enhancement was observed with incubation of BV-2 cells with the four mitochondrial respiratory inhibitors, and reduced by NAC, which confirms that these four respiratory inhibitors of the respiratory chain result in outbursts of HOCl (Fig. 3B). Then, the mitochondrial membrane potential (Δ*Ψ*_m_) as an indicator of mitochondrial function, was used to evaluate the origin of HOCl. In the subsequent research, we measured changes in Δ*Ψ*_m_ using the red/green fluorescence ratio (JC-1 assay, Fig. 3C and E). The same trend as Fig. 3A was observed, indicating that the destruction of the cell respiratory chain may damage the cell mitochondria, resulting in the formation of HOCl. In addition, confocal imaging indicated that maximal HOCl production was induced by antimycin A (Fig. 3A and D), which prompted us to determine the dynamic real-time monitoring ability of antimycin A (5 μM) to produce HOCl. As shown in Fig. 3G, H and S20,† the fluorescence signals of both groups appeared in the first 5 min and increased gradually for the subsequent 10 min (5–15 min). This phenomenon indicates that the probe fully entered the cell after 5 min and that HOCl production began after 5 min. In addition, compared with the antimycin A + APO (apocynin, 10 mM, NADPH oxidase 2 inhibitor^20^) group, a significant increase in *I*_red_ was observed for antimycin A, indicating that the fluorescence signal changes during antimycin A-induced HOCl production were mainly attributed to changes in oxidative stress.

Strokes are usually caused by the natural reperfusion or iatrogenic reperfusion of ischemic brain tissue, which further damages the structure and functional metabolism of the nervous system, *i.e.*, cerebral ischemia-reperfusion injury (CIRI, which results from prolonged ischemic insult followed by the restoration of blood perfusion, which can induce tissue damage and initiate a cascade of deleterious cellular responses, leading to cell death and ultimately organ failure. Under hypoxia, cells consume several intracellular reducing substances, especially reductases, to maintain normal metabolic activity. During reperfusion, the ROS byproducts produced by mitochondria when the cells return to normal respiration cannot be cleared sufficiently quickly, resulting in oxidative stress damage to cells and tissues).^21^ Based on the above experimental results, we speculate that HOCl can be used to characterize CIRI, however this connection remains largely unexplored because of the strong oxidizing properties, short lifetime, and low concentration of HOCl during this process.^22^ To confirm this assumption, WD-HOCl was used to monitor HOCl changes with real-time tracking (0–60 min) during oxygen–glucose deprivation/reperfusion (OGD/R) to simulate the occurrence of CIRI in cells. Briefly, cells were kept in sugar-free DMEM and a three-gas incubator for 12 h without oxygen, and then the normal state of cultured cells was resumed. As presented in Fig. 3G, H and S20,† the relative fluorescence intensity increased dramatically upon increasing the OGD/R time, indicating that the HOCl content gradually increased upon prolonging the OGD/R. To further confirm the source of HOCl during the OGD/R process, we used APO, which can reduce ROS generation, in an attempt to ameliorate cell death and cerebral injuries induced by CIRI. Cells were incubated with APO (10 mM) and subjected to OGD/R over different time periods. Compared with the OGD/R group, the fluorescence signals were reduced after the cells were co-incubated with APO. This suggests that APO can reduce ROS production during CIRI and therefore inhibit apoptosis caused by oxidative stress (Fig. 3F).

**Fig. 4:**
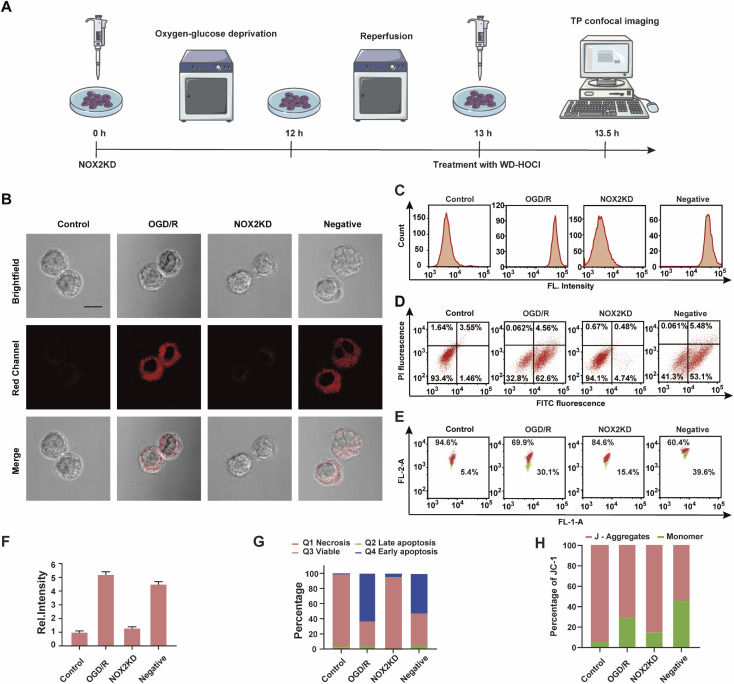
(A) Schematic illustration of the OGD/R modelling and probe treatment; (B) imaging of WD-HOCl-loaded BV-2 cells. Control, untreated cells; ODG/R, OGD/R treated cells; NOX2KD, NADPH oxidase 2 gene knockout; negative, negative control of NOX2KD. *n* = 48, 61, 55 and 65 cells, respectively. Emissions were collected in the red channel (680–738 nm) with 606 nm excitation. Scale bar: 20 μm. (C) Flow cytometry analysis of (B); (D) apoptosis assay of cells in (B); viable cells (FITC−/PI−), early apoptosis (FITC+/PI−), late apoptosis, and necrosis (FITC+/PI+); (E) mitochondrial membrane potential analyzed by JC-1; (F) histograms of the average fluorescent intensities in (B); (G) quantification data of the apoptosis assay results in (D); (H) quantification data of JC-1 assay results in (E). In (F)–(H), error bars represent SD.

NOX2 is a key enzyme that produces cellular reactive oxygen species (ROS) that can induce oxidative stress. To further elucidate the relationship between oxidative stress and cerebral strokes, the NOX2 gene of BV-2 cells was removed (NOX2KD, operational approach according to the reported methods^23^) and cultured in a sugar-free and oxygen-free environment for 12 h (Fig. 4A). Then, the cells were incubated in normal medium for 1 h and then incubated with WD-HOCl. Fig. 4B and F indicated that the *I*_red_ value obtained from the OGD/R group was much higher than that from the control group, but the *I*_red_ value of the BV-2 cells changed slightly after removal of the NOX2 gene (NOX2KD). The *I*_red_ value of the negative control group (performed according to a previous report^23^) was significantly enhanced. Flow cytometric analysis also indicated changes in the fluorescence intensity, confirming that the OGD/R process results in oxidative stress of the cells (Fig. 4C). Apoptosis in these processes was assayed using an Annexin V-FITC/PI apoptosis assay kit, as shown in Fig. 4D and G. Cells in the control and NOX2KD groups maintained similar and high survival rates, but the apoptosis rates of cells in the OGD/R and negative groups were 62.6% and 53.1% respectively and the cell survival rates were only 32.8 and 41.3%. These results suggest that the oxidative stress levels in the OGD/R cells can regulate the degree of apoptosis. Δ*Ψ*_m_ was also measured using the JC-1 assay, and the results indicated that the change was consistent with that of the Annexin V-FITC/PI apoptosis assay, confirming that apoptosis induced by OGD/R was caused by mitochondrial damage (Fig. 4E and H). The released ROS could induce the overexpression of the inflammatory vesicle, NLRP3 (NOD-like receptor thermal protein domain associated protein 3) and COX-2 (cyclooxygenase 2), which results in cellular inflammation. The above process also induced significant oxidative stress. Therefore, the expression levels of the cellular oxidative stress marker (NOX2) and inflammatory factors (NLRP3 and COX-2) in the above process were evaluated. As shown in Fig. S21,† the concentration trends of the mentioned proteins in cells under different treatments were detected using western blotting, and the relationship between oxidative stress and inflammatory response during OGD/R was confirmed.

### *In vivo* imaging of WD-HOCl during stroke

**Fig. 5:**
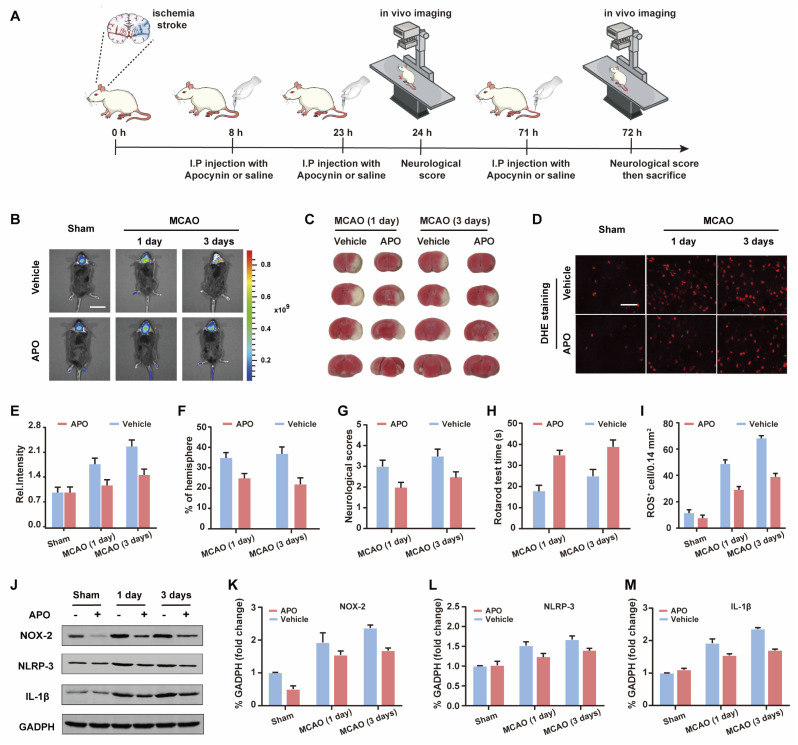
(A) Schematic illustration of the middle cerebral artery occlusion (MCAO) modeling method and probe treatment. (B) *In vivo* imaging of HOCl in the brain during middle cerebral artery occlusion (MCAO) at different times when subjected to different treatments: sham group (mice not undergoing MCAO), MCAO group (mice undergoing MCAO), vehicle group (injection of saline to mice tail veins), and APO (2.0 mg kg^−1^) group (intraperitoneally injection with apocynin in mice). Emissions were collected at the red channel (680–738 nm) with 606 nm excitation. Scale bar = 2 cm. (C) 2,3,5-Triphenyltetrazolium chloride (TTC) staining images of infarct regions in slices of the ipsilateral hemisphere undergoing different treatments. (D) ROS revealed by dihydroethidium (DHE) staining from samples in panel (B). (E) Histogram of the average fluorescence intensities in panel (B). (F) Percentages of infarct size for the contralateral hemispheres shown in (B). (G) Rotarod test. (H) Neurological scores. (I) Quantification of ROS-positive cells in the ischemic border. Scale bar, 50 μm. (J) Western blotting illustrating the expression of NOX-2, NLRP-3, IL-1β, and GAPDH from the samples in (B); quantification data of the western blot results of NOX-2 (K), NLRP-3 (L), and IL-1β (M) from the samples in (J). In (E)–(I), and (K)–(M), the error bars indicate the SD.

Based on the good results observed for the cell OGD/R models, we decided to use WD-HOCl to monitor HOCl fluctuations during *in vivo* stroke processes. The *in vivo* cytotoxicity of WD-HOCl was evaluated using the hematoxylin and eosin staining (H&E) of organs after the intravenous injection of the probe. As shown in Fig. S22,† no necrosis, edema, inflammatory infiltration, or hyperplasia were observed in any of the tissues, indicating that WD-HOCl (200 μL, 200 μM) exhibited good biocompatibility and could be used for *in vivo* imaging. Combined with 2,3,5-triphenyltetrazolium chloride (TTC, measures tissue viability and is used to evaluate the infarct size) staining (Fig. 5C and F), the mouse cerebral stroke model was established using the middle cerebral artery occlusion (MCAO) method. *In situ* detection results indicated that the *I*_red_ values 1 and 3 days after a cerebral stroke were significantly higher than the 0 day group. This indicates that the probe can monitor oxidative stress during a cerebral stroke *in vivo* (Fig. 5B and C). However, the fluorescence intensity of mice after intraperitoneal injection of APO (2.0 mg kg^−1^) was almost unchanged, which again suggests that APO can inhibit brain oxidative stress and reduce the concentration of HOCl in mice. Additionally, the rotarod test was used to evaluate the effect of APO on the motor coordination of mice and results similar to the neurological scores, with both behavioral tests indicated that APO improved behavioral performance after 3 days (Fig. 5G and H). In addition, we evaluated the NOX2 expression and ROS levels using a western blot and DHE staining, respectively (Fig. 5D and I–K). The results indicated that the production of both NOX2 and ROS were significantly higher three days after a stroke. Furthermore, APO injection significantly suppressed the production of ROS while also inhibiting NOX2 expression. The western blot results indicated that the inflammatory-related proteins, NLRP3 and IL-1β (interleukin-1 beta), exhibited similar expression levels 3 days after a stroke and were significantly inhibited by APO. These results confirm that the onset of stroke was accompanied by neuroinflammation (Fig. 5J, L and M).

## Conclusions

With this research we have developed a NIR fluorescent probe WD-HOCl that exhibits a large Stokes shift of 156 nm, which enables almost complete separation of its excitation and emission peaks. The probe was able to target mitochondria and was suitable for bioimaging and the evaluation of HOCl levels during stroke. WD-HOCl rapidly reacted with HClO and could be used for the selective and sensitive detection of HOCl without interference from other ROS or RNS. WD-HOCl specifically accumulated in the mitochondria and highlighted changes in endogenous HOCl levels. Using cell OGD/R models, WD-HOCl was used to monitor HOCl levels. Based on HOCl imaging in a mouse stroke model using WD-HOCl, we were able to determine that a stroke causes extreme damage to brain tissue in the early stage, and APO can reduce the production of HOCl by inhibiting oxidative stress and neuroinflammation *in vivo*. In summary, WD-HOCl is a promising and effective tool for bioimaging and medical diagnosis.

## Ethical statement

Wild-type C57BL/6J mice (*n* = 300; 25–30 g) were purchased from Hubei Experimental Animal Research Center (Hubei, China; no. 43004700018817, 43004700020932). All animal experimental protocols were approved by the Animal Experimentation Ethics Committee of South-Central University for Nationalities (No. 2020-scuec-043) and were conducted according to the Animal Care and Use Committee guidelines of South-Central University for Nationalities. Animals were housed in a room with controlled humidity (65 ± 5%) and temperature (25 ± 1 °C), under a 12/12 hour light/dark cycle with free access to food and water for at least 1 week before the experiments.

## Data availability

The datasets supporting this article have been uploaded as part of the ESI.†

## Author contributions

W. Hu, T. Qiang and T. D. James conceived the project. W. Hu, F. Cheng and X. Song synthesized the dyes and conducted photophysical characterization and analyses. W. Hu, C. Li and B. Wang performed fluorescence imaging experiments. L. Ren, and M. Li preformed the OGD/R and MCAO experiments. T. Qiang characterized all the compounds. All authors participated in writing the manuscript.

## Conflicts of interest

TDJ acts as an academic consultant for TQ as part of a guest professorship at SUST.

## Supplementary Material

SC-013-D2SC03833A-s001
