# Supplementary material for: Imaging of hypochlorous acid in mitochondria using an asymmetric near-infrared fluorescent probe with large Stokes shift
Source: Chem Sci. 2022 Sep 8;13(37):11140–9. doi: 10.1039/d2sc03833a (PMC9516895; doi:10.1039/d2sc03833a)
Supplement: SC-013-D2SC03833A-s001 [file SC-013-D2SC03833A-s001.pdf]

Electronic Supplementary Material (ESI) for Chemical Science.
This journal is © The Royal Society of Chemistry 2022

Supporting Information

Imaging of Hypochlorous Acid in Mitochondria using an Asymmetric Near-Infrared Fluorescent Probe with Large Stokes Shift

Wei Hu [a, c], Taotao Qiang [a, *], Chenchen Li [b], Longfang Ren [a], Fei Cheng [a], Baoshuai Wang [a], Mingli Li [a], Xinjian Song [b, *], Tony D. James [c,d,*]

[a] *College of Bioresources and Materials Engineering, Shaanxi Collaborative Innovation Center of Industrial Auxiliary Chemistry & Technology, Shaanxi University of Science & Technology, Xi'an, 710021, China.*

[b] *Hubei Key Laboratory of Biological Resources Protection and Utilization, Hubei University for Nationalities, Enshi, 445000, China.*

[c] *Department of Chemistry, University of Bath, Bath, BA27AY, UK.*

[d] *School of Chemistry and Chemical Engineering, Henan Normal University, Xinxiang 453007, China.*

*Corresponding author.

E-mail: t.d.james@bath.ac.uk (Tony D. James), qiangtt515@163.com (Taotao Qiang) and whxjsong@163.com. (Xinjian Song).

Table of Contents

1. General Information on Materials and Methods. S3
Instruments and materials.. S3
Spectroscopic measurements.. S3
Determination of the detection limit. S4
Quantum yield measurements. S4
DFT calculations. S4
Water solubility assay. S5
Cytotoxicity Assay. S5
Cell Culture and Imaging. S5
Histological Staining of the Tissue Slices. S5
Calculation of Mean Fluorescence Intensity. S6
Western blotting assay. S6
OGD/R model. S6
Flow Cytometry Analysis. S7
Measurement of ROS. S7
Middle cerebral artery occlusion (MCAO) model. S7
Measurement of infarct volume and neurological deficit. S8
Assessment of neurological deficit. S8
Rotating beam walking test. S8
Rotarod test. S9
In vivo imaging studies.. S9
2. Synthesis of **WD-HOCl**. S10
3. Supplementary Figures S12
4. Supplementary Table S35
5. References.. S36

1. General Information on Materials and Methods.

Instruments and materials.

Unless otherwise stated, all solvents and reagents were purchased from commercial suppliers and were used as received without further purification. PC12 cells and BV-2 cells were obtained from Procell Life Science & Technology Co., Ltd. 3-(4,5-Dimethylthiazol-2-yl)-2,5-diphenyltetrazolium bromide (MTT), Green commercial co-localization dyes (Lyso-Tracker Green for lysosomes, Mito-Tracker Green for mitochondria, and ER-Tracker Green for the endoplasmic reticulum), phorbol 12-myristate-13-acetate, Lipoic acid, N-acetyl-L-cysteine, L-methionine, 3-Methyladenine, Rotenone, thenoyltrifluoroacetone, KCN, Antimycin A, JC-1 and Apocynin were purchased from Sigma-Aldrich. Absorption spectra were recorded on a UV-Vis spectrophotometer (Shimadzu UV-2550, Japan), and one-photon fluorescence spectra were obtained using a fluorimeter (Shimadzu RF-6000, Japan) using a 1 cm standard quartz cuvette. ^{1}H and ^{13}C NMR spectra were recorded on a Bruker-400 spectrometer, using TMS as an internal standard. Flow cytometric data were collected by BD Biosciences Facsaria. Confocal fluorescence imaging of fluorescently stained cells was performed on a confocal laser scanning microscope (Carl Zeiss, Germany). The product of the probe was detected by liquid chromatography-tandem mass spectrometry (LC/MS, Agilent 1290 Infinity II-6230 TOF). Fluorescence imaging of mice was performed on an IVIS Lumina LT Series III small animal optical *in vivo* imaging system (U.S.A.) with an excitation filter of 600 nm and an emission filter of 710 nm. Experimental mice were anesthetized on an R500IE anesthesia machine. Living Image 4.5 software (PerkinElmer) was used for data analysis.

Spectroscopic measurements.

Unless otherwise noted, all the measurements using **WD-HOCl** were evaluated in PBS buffer (10 mM, pH 7.4, containing 33.3% acetonitrile). After adding NaOCl and incubating at 37 °C for 5 min, a 500 μL aliquot of the reaction solution was transferred to a quartz cell with an optical length of 1 cm for the measurement of absorbance or fluorescence. The excitation wavelengths was 606 nm.

For the selectivity assay, superoxide anion ($O_2^{\bullet-}$) was created by the enzymatic reaction of xanthine/xanthine oxidase (XA/XO; 6.0 μM/3 mU) at 25 °C for 5 min[1-3].

•OH was generated by Fenton reaction between Fe^{2+} (EDTA) and H_2O_2 quantitively, and Fe^{2+} (EDTA) concentrations represented •OH concentrations[4]. The $ONOO^-$ source was the donor 3-Morpholinosydnonimine hydrochloride (SIN-1, 100 μM)[5]. NO was generated from 3-(aminopropyl)-1-hydroxy-3-isopropyl-2-oxo-1-triazene (NOC-5, 100 μM)[6]. NO_2^- was generated from $NaNO_2$. OCl^- was standardized at pH 12 (ε_{292} nm = 350 $M^{-1}cm^{-1}$) [7,8]. The H_2O_2 concentation was determined at 240 nm ($\varepsilon_{240\ nm}$ = 43.6 $M^{-1}cm^{-1}$). All the reagents were obtained from Aladdin (USA). All other chemicals were from commercial sources and of analytical reagent grade unless otherwise indicated.

Determination of the detection limit.

The limit of detection (LOD) for HOCl was calculated based on the following equation[9]:

$$LOD = 3\sigma/k$$

Where σ represents the standard deviation and k represents the slope of the titration spectra curve among the limited range.

Quantum yield measurements.

The measurement of the fluorescence quantum yield was measured by using an ethanol solution of rhodamine B as the standard (10 μM, Φ_r= 0.71) and using the following equation[9].

$$\Phi_s = (A_r \cdot F_s \cdot n_s^2)/ (A_s \cdot F_r \cdot n_r^2)\ \Phi_r\ (A \leq 0.05)$$

Where s and r represent the sample to be tested and the reference dye, respectively. A represents the absorbance at the maximum absorption wavelength, F represents the fluorescence spectrum integral at the maximum absorption wavelength, and n represents the refractive index of the sample to be tested or the reference dye solvent.

DFT calculations.

All the calculations were carried out using the Gaussian 09 program package[10]. The ground state (S_0) and singlet excited state (S_1) of the dyes are calculated with

density functional theory (DFT) and time-dependent DFT (TD-DFT) at B3LYP/6-31G(d) level using a PCM solvation model with EtOH as the solvent. The molecular orbital (MO) plots and MO energy levels are computed at the same level of theory. The absorption and emission wavelengths are calculated by TD-DFT methodology using the same basis set based on the optimized S_0 and S_1 geometries.

Water solubility assay.

A small amount of **WD-HOCl** was dissolved in acetonitrile to prepare the stock solutions (0.01 M). In all cases, the concentration of acetonitrile was maintained to be 33%. The plot of fluorescence intensity against the **WD-HOCl** concentration was linear at low concentrations and exhibited downward curvature at higher concentrations, and the maximum concentration in the linear region was taken as the solubility.

Cytotoxicity Assay.

MTT tests were performed with reference to the reported protocol[11] with a minor change. BV-2 cells were seeded in 96-well plates and incubated with different concentrations of **WD-HOCl** (0, 2, 5, 10 and 15 μM, containing 1% DMSO in 200 μL DMEM). The experiment and control groups were incubated in an atmosphere of 5/95 (v/v) of CO_2/air at 37 °C for 24 h. Next, 20 μL 5.0 mg/mL MTT solution was added into each well, followed by incubation for 4 h under the same conditions. Then the 100 μL supernatant were removed and 150 μL DMSO added. After shaking for 10 min, the absorbance at 490 nm was measured by microplate reader (Synergy 2, BioTek Instruments Inc.). Cell survival rate was calculated by $A/A_0 \times 100\%$ (A and A_0 are the absorbance of the experimental group and control group, respectively).

Cell Culture and Imaging.

BV-2 and PC12 cells were cultured in Dulbecco's modified Eagle's medium (DMEM, Thermo Scientific) supplemented with 1% penicillin/streptomycin and 10% fetal bovine serum (FBS), and incubated in an atmosphere of 5/95 (v/v) of CO_2/air at 37 °C. Two days before imaging, the cells were passed and placed into glass-bottomed dishes (NEST). For labeling, the cells were washed with serum-free DMEM and then

incubated with 5 μM **WD-HOCl** (containing 1% DMSO) for 30 min at 37 °C.

Histological Staining of the Tissue Slices.

After imaging, the mice were killed, and the livers were collected for tissue analysis. Through a series of standard procedures, including fixation in 10% neutral buffered formalin, embedding into paraffin and sectioning at 3 μm thickness, the tissues were stained with hematoxylin-eosin (H&E). Thereafter, the prepared slices were examined using a digital microscope.

Calculation of Mean Fluorescence Intensity.

The mean fluorescence density was measured by Image-Pro Plus (v. 6.0) and calculated *via* the equation (mean density = $IOD_{sum}/area_{sum}$), where IOD and area were integral optical density and area of fluorescent region.

Western blotting assay.

Western blotting was carried out as previously reported[12]. Cortical sections 1.0 to 2.0 mm from ipsilateral brain tissue was harvested and homogenized in cold RIPA buffer (C1053, Applygen, Beijing, China) plus protease inhibitor cocktail (G2006, Servicebio, Wuhan, China). The homogenates were centrifuged at 4 °C at 10, 000 × g for 30 min, and then the supernatants were harvested. Protein content was determined with the BCA kit (G2026, Servicebio, Wuhan, China). Protein samples (20 μL/lane) were separated by electrophoresis on 4-15% sodium dodecyl sulfate-polyacrylamide gels and then transferred onto PVDF membranes (Millipore, Billerica, MA, USA). Membranes were then put into 5% non-fat milk with PBS/0.1% Tween and blocked for 1 h, followed by incubation overnight with mouse anti-NLPR3 (1:1,000; ab4207, Cell Signaling Technology, Boston, USA), anti-NOX2 (1:1,000; ab18256, Abcam, Cambridge, England), anti-IL-1β (1:1,000; ab12703, Cell Signaling Technology, Boston, USA) and anti-COX-2 (1:1,000; 4842, Cell Signaling Technology, Boston, USA) at 4 °C. After washing with PBS/0.1% Tween, the membrane was incubated with IR Dye-labeled secondary antibody (1:10,000; c60405-05, Li-Cor Bioscience, USA) at room temperature for 1–2 h. Images were acquired with the Odyssey Western Blot

Analysis system (LI-COR, Lincoln, NE, USA). The relative band intensity was calculated using Quantity One v4.6.2 software (Bio-Rad Laboratories, Hercules, USA) and then normalized to the GADPH loading control. All the above experiments were repeated three times.

OGD/R model.

OGD/R model of cells was performed by oxygen and glucose deprivation/reperfusion. BV-2 cells at 80% confluence were harvested by scraping and transferred to confocal dishes to grow[13]. When the cells are adherent, the culture medium is changed to sugar-free DMEM and cultured in a three-gas incubator for 12 hours without oxygen. Afterwards, these cells were incubated with high-glucose DMEM in a 5 % CO_2 and 95% O_2 atmosphere for different times. Then, the cells were incubated with **WD-HOCl** for 30 min and washed with PBS for three times to perform confocal imaging.

Flow Cytometry Analysis.

The BV-2 cells were cultured at 1.0×10^6 cells/well in 6-well plates, and then treated with different treatment as described in the paper. After harvest, cells were washed and suspended in fresh complete medium. 5 μL AnnexinV-FITC was mixed, then add 5 μL PI mix and analyzed by flow cytometry.

Measurement of ROS.

To assess ROS production, the brain was carefully and quickly isolated and cut into 4.0 μm sections and placed on chilled microscope slides. The samples were incubated in physiological saline solution containing 10 μmol dihydroethidium (DHE; Sigma-Aldrich) for 30 min at 37 °C in the dark room. The brain was washed twice with PSS and placed under automatic fluorescence microscope (BX63, Olympus Optical Ltd, Tokyo, Japan).

Middle cerebral artery occlusion (MCAO) model.

Wild-type C57BL/6J mice (n = 300; 25–30 g) were purchased from Hubei

Experimental Animal Research Center. (Hubei, China; No. 43004700018817, 43004700020932). All animal experimental protocols were approved by the Animal Experimentation Ethics Committee of South-Central University for Nationalities (No. 2020-scuec-043) and were conducted according to the Animal Care and Use Committee guidelines of South-Central University for Nationalities. Animals were housed in a room with controlled humidity (65 ± 5%) and temperature (25 ± 1 °C), under a 12/12-hour light/dark cycle with free access to food and water for at least 1 week before the experiments. MCAO was induced using a previously described method with slight modifications[14]. In brief, C57BL/6J wild-type mice were anesthetized with 5% isoflurane in O_2 by facemask, followed by ligation of the left middle cerebral artery with 6-0 monofilament (Doccol Corp., Redlands, CA, USA). After 1 h of occlusion, the monofilament was removed to initiate reperfusion. A homeothermic heating pad was employed to monitor and stabilize the mice body temperature at 37 ± 0.5 °C. The same procedure, but without monofilament ligation, was performed on sham-operated mice.

Measurement of infarct volume and neurological deficit.

Wild-type C57BL/6J mice mentioned above were deeply anesthetized and euthanized with an overdose of isoflurane and decapitated 1 and 3 days after MCAO (i.e., after 3days of reperfusion). The brains were collected after transcranial perfusion by saline followed with 4% paraformaldehyde. Brain tissues were cut into 1-mm coronal sections, and then dipped in 2% 2,3,5-triphenyltetrazolium chloride (TTC) (17779, Sigma-Aldrich, United States) for staining. The infarct volume was measured and analyzed by a blinded observer using ImageJ v1.37 (NIH, Bethesda, MA, United States), as described previously[15-17], then normalized and presented as a percentage of the non- ischemic hemisphere to correct for edema[18]. Neurological deficit scores were evaluated 3 days after MCAO as described previously[19]. The score ranged from 0 (without observable neurological deficit) to 4 (no spontaneous motor activity and loss of consciousness).

Assessment of neurological deficit.

Neurological deficit scores were evaluated 3, 7 and 14 days after MCAO as described previously[15]. The score ranged from 0 (without observable neurological deficit) to 4 (no spontaneous motor activity and loss of consciousness).

Rotating beam walking test.

The rotating beam walking test was used to evaluate neurological deficits in coordination and integration of movement in mice after MCAO[20]. The mice were trained to walk along a 100 cm rotating wood beam (80 mm in diameter, approximately 80 cm above the floor, at 3 rpm rotation) for 3 days (3 trials per day), then tested before and at 1 and 3 days after stroke. The walking time at each time point for each mouse was then recorded.

Rotarod test.

Accelerating rotarod (SD Instruments, San Diego, CA) Instruments were used to test the motor coordination ability of the mice. Each MCAO model mouse was placed on a 2.75 cm diameter rotating rod every other day before MCAO onset for a total of 9 training days, and the rotation speed of rod increased from 5 to 10 rpm every 5 min. The time between the beginning of mouse staying on the rod and falling from the rod was determined up to a maximum duration of 300 seconds. After training period, MCAO surgery was conducted and Rotarod testing was performed by a blinded observer on 0, 1, 3 day post-stroke. The scores were calculated by averaging the three repetitive times records of each mouse each day.

***In vivo* imaging studies**

After the MCAO model of Wild-type C57BL/6J mice mentioned above was successfully established, **WD-HOCl** (200 μL, 200 μM) was injected through the tail vein, and the mice were anesthetized with isoflurane before fluorescence imaging using a Bruker *in vivo* imaging system. Whereafter, the mice were anesthetized and dissected to remove the mouse brain tissue, and a 300 μm section was prepared with a microtome.

Two-photon excited tissue fluorescence images were obtained by Zeiss LSM 710 multiphoton laser scanning confocal microscope.

2. Synthesis of WD-HOCl.

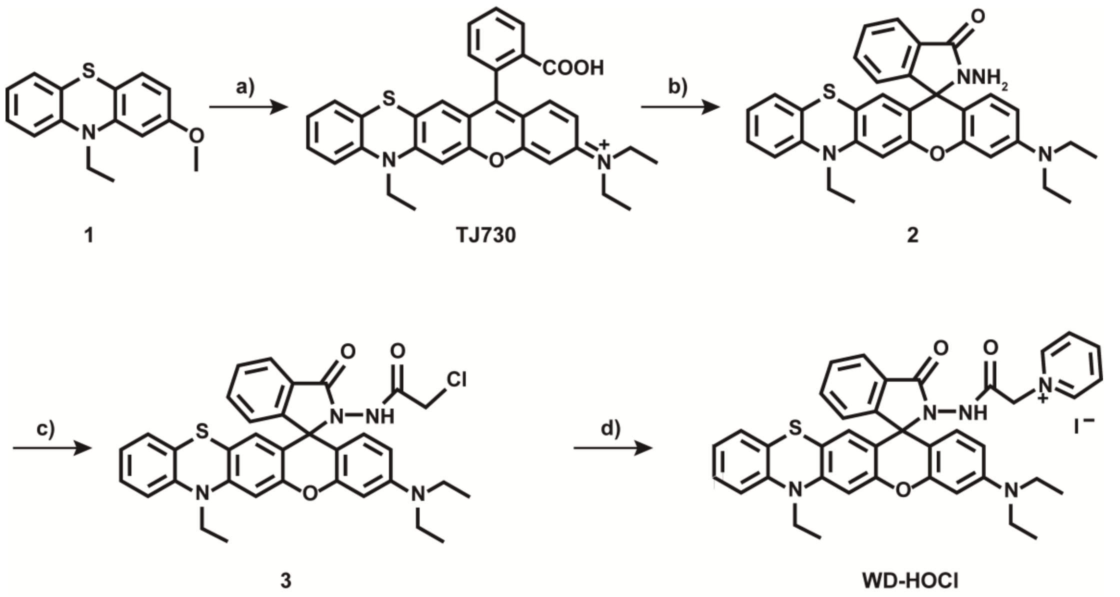

Scheme S1 Synthetic route of **WD-HOCl**. Reagents and conditions: (a) 2-(4-(Diethylamino)-2-hydroxy-benzoyl)benzoic acid, methanesulfonic acid, 70 °C, 12 h. (b) MeOH, hydrazine hydrate, 80 °C for 12 h. (c) 2-Chloroacetyl chloride, triethylamine, dry chloroform, r.t. for 4 h. (d) Pyridine, KI, MeCN, 80 °C for 24 h.

Synthesis. The synthetic route for compound **WD-HOCl** from commercially available materials is depicted in Scheme S1 and Compound **TJ730** was prepared according to previous report[21].

Compound 2: **TJ730** (1.041 g, 2.0 mmol) and hydrazine (85%, 588.9 mg, 10.0 mmol) was dissolved in MeOH (15 mL) and 80 °C for 12 h. After the completion of the reaction, the solvent was removed and the crude residue was purified by column chromatography (1:2 v/v ethyl acetate/dichloromethane) to give compound **2** as a white solid. Yield: 886.44 mg (83%). ^{1}H NMR (400 MHz, $CDCl_3$; Fig. S1) δ 7.99–7.95 (m, 1H), 7.52–7.47 (m, 2H), 7.16 (td, J = 8.0, 1.3 Hz, 1H), 7.12–7.03 (m, 2H), 6.91 (t, J = 7.4 Hz, 2H), 6.73 (s, 1H), 6.47–6.43 (m, 2H), 6.39 (s, 1H), 6.33 (dd, J = 8.8, 2.4 Hz, 1H), 3.97 (q, J = 6.9 Hz, 2H), 3.71 (s, 2H), 3.36 (q, J = 7.0 Hz, 4H), 1.49 (t, J = 6.9 Hz, 3H), 1.19 (t, J = 7.0 Hz, 6H). ^{13}C NMR (100 MHz, $CDCl_3$; Fig. S2) δ 166.25, 153.45, 152.49, 150.90, 148.98, 146.32, 143.81, 132.78, 129.79, 128.53, 128.03, 127.37, 127.31, 125.09, 123.84, 123.80, 123.18, 122.67, 118.85, 115.20, 112.48, 108.42, 104.03, 103.30, 97.89,

65.39, 44.39, 42.08, 12.79, 12.59. HR-MS (MALDI-DHB; Fig. S3) calcd. for $C_{32}H_{30}N_4O_2S$ $[M+H]^+$: 535.2168, found: 535.2138.

Compound 3: To a solution of compound **2** (802.0 mg, 1.5 mmol) in dry chloroform (10 mL), NEt_3 (202.4 mg, 2.0 mmol), chloroacetyl chloride (225.9 mg, 2.0 mmol) containing 2 mL of dry chloroform were successively added under stirring below 5 °C. The mixture was warmed to r.t. and stirred for 4 h, then poured into cold water and extracted with dichloromethane. The combined organic phase was washed with brine and dried over anhydrous Na_2SO_4. After removing solvent, the residue was purified by column chromatography to give compound **3** as white solid. Yield: 722.85 mg (79%). ^{1}H NMR (400 MHz, $CDCl_3$; Fig. S4) δ 7.93 (dd, J = 8.7, 6.7 Hz, 1H), 7.62 (s, 1H), 7.45 (m, 2H), 7.05 (dd, J = 8.4, 7.8 Hz, 2H), 6.96 (d, J = 7.5 Hz, 1H), 6.81 (t, J = 7.8 Hz, 2H), 6.58 (t, J = 7.5 Hz, 2H), 6.45 (s, 1H), 6.27 (dd, J = 11.4, 9.8 Hz, 2H), 3.87 (d, J = 8.5 Hz, 4H), 3.44–3.05 (m, 4H), 1.40 (t, J = 6.8 Hz, 3H), 1.09 (t, J = 6.8 Hz, 6H). ^{13}C NMR (101 MHz, $CDCl_3$; Fig. S5) δ 163.61, 162.82, 152.41, 151.41, 149.72, 148.18, 145.46, 142.60, 132.57, 128.36, 127.77, 127.64, 126.37, 126.25, 124.98, 123.10, 122.80, 121.66, 117.70, 114.14, 110.55, 107.38, 102.07, 101.84, 97.15, 96.41, 64.59, 43.35, 41.13, 40.08, 11.71, 11.56. HR-MS (MALDI-DHB; Fig. S6) calcd. for $C_{34}H_{31}ClN_4O_3S$ $[M+H]^+$: 611.1884, found: 611.1834.

Probe WD-HOCl: Compound **3** (305.6 mg, 0.5 mmol) was dissolved in dry acetonitrile. Pyridine (395 mg, 5 mmol) and KI (166 mg, 1 mmol) were added and refluxed for 24 h. The reaction solvent was removed under vacuum, and the residue was further purified by column chromatography to give **WD-HOCl** as white solid. Yield: 238.21 mg (61%). ^{1}H NMR (400 MHz, $CDCl_3$; Fig. S7) δ 9.94 (s, 1H), 8.80 (d, J = 5.4 Hz, 2H), 8.28 (t, J = 7.6 Hz, 1H), 7.77 (d, J = 6.9 Hz, 3H), 7.51–7.39 (m, 2H), 7.12–6.97 (m, 2H), 6.89–6.73 (m, 3H), 6.66 (s, 1H), 6.56 (d, J = 8.9 Hz, 1H), 6.45 (s, 1H), 6.36 (s, 1H), 6.22 (d, J = 8.5 Hz, 1H), 5.65 (dd, J = 39.4, 15.3 Hz, 2H), 3.89 (d, J = 6.7 Hz, 2H), 3.26 (d, J = 6.9 Hz, 4H), 1.40 (t, J = 6.9 Hz, 3H), 1.09 (t, J = 6.9 Hz, 6H). ^{13}C NMR (101 MHz, $CDCl_3$; Fig. S8) δ 163.69, 161.79, 152.26, 151.42, 149.95, 148.18, 145.21, 144.84, 144.69, 142.60, 132.72, 128.29, 127.83, 127.08, 126.63, 126.30, 126.24, 125.35, 123.13, 122.69, 122.62, 121.55, 116.95, 114.25, 110.10, 107.54, 102.26, 101.89, 96.85, 65.13, 60.05, 43.44, 41.28, 11.81, 11.69. HR-MS (MALDI-DHB; Fig. S9) calcd. for $C_{39}H_{36}N_5O_3S^+$ $[M]^+$: 654.2533, found: 654.2501.

3. Supplementary Figures

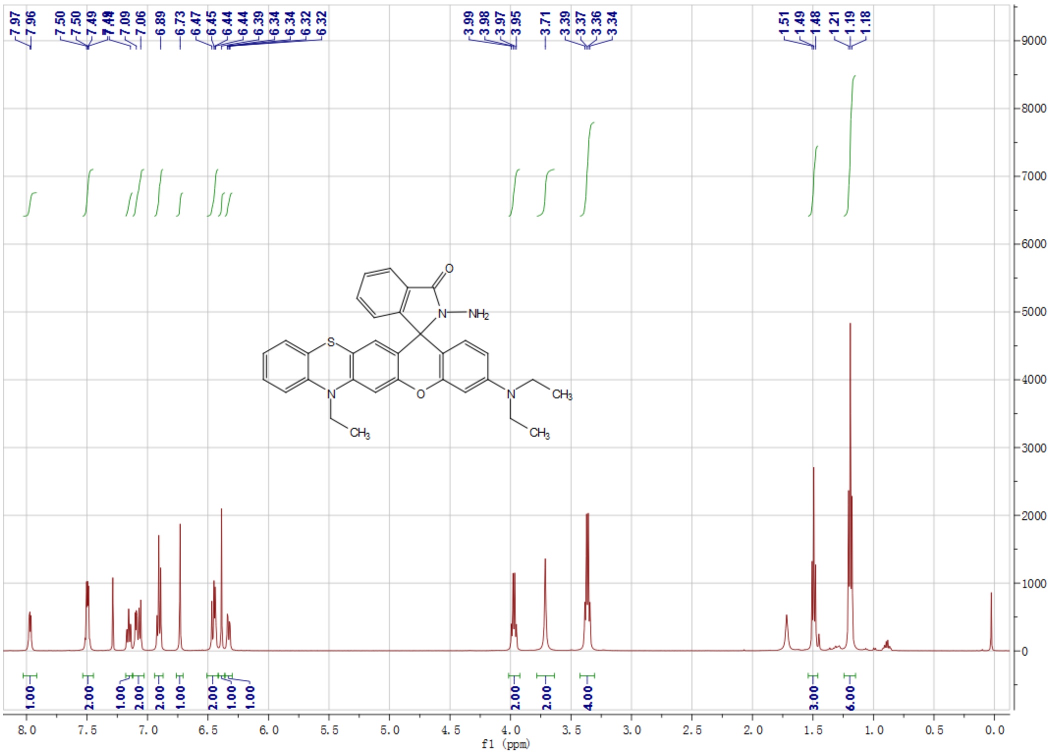

Fig. S1. ^1H NMR spectrum (400 MHz, $CDCl_3$) of compound **2**.

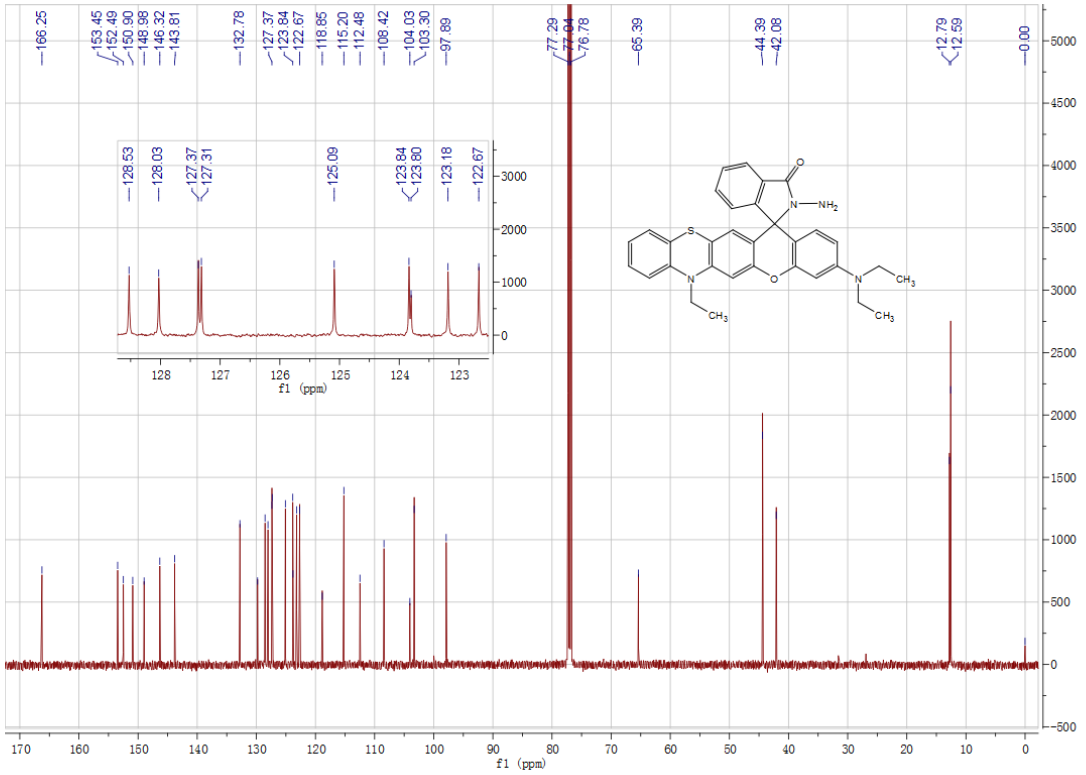

Fig. S2. ^{13}C NMR spectrum (100 MHz, $CDCl_3$) of compound **2**.

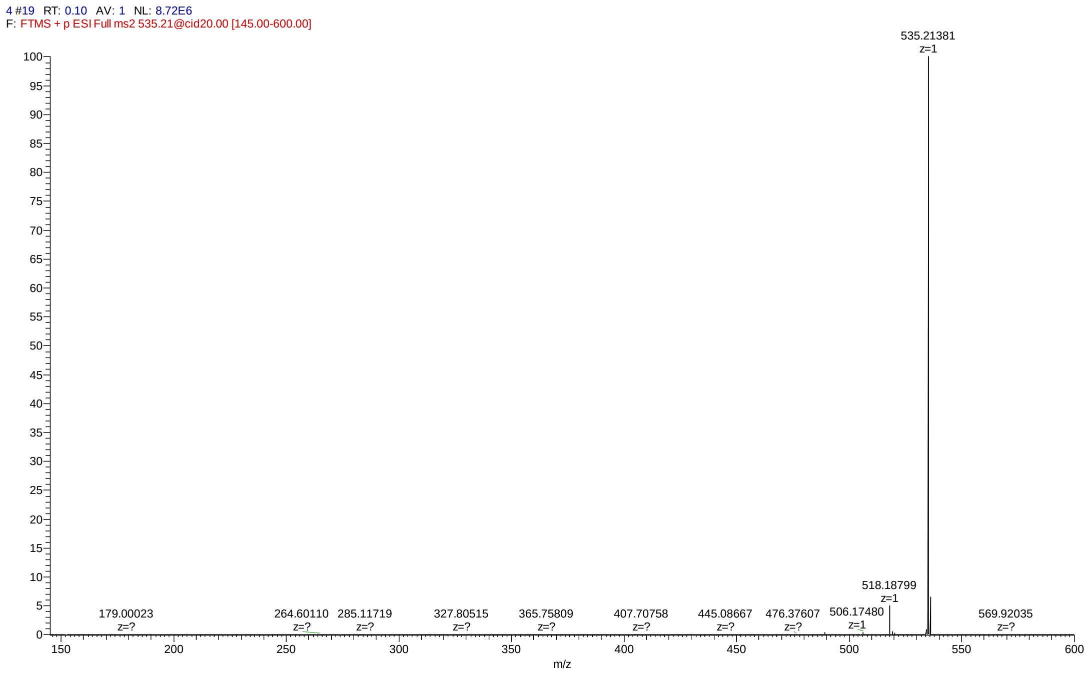

Fig. S3. HR-MS spectrum of compound 2.

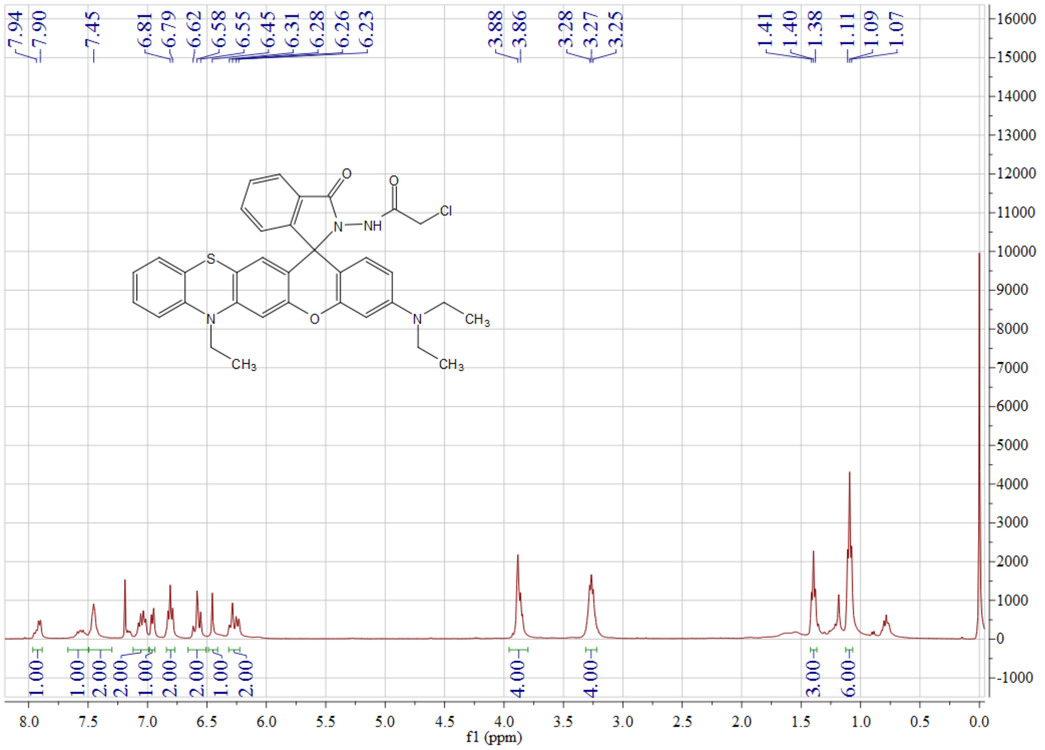

Fig. S4. ^{1}H NMR spectrum (400 MHz, $CDCl_3$) of compound **3**.

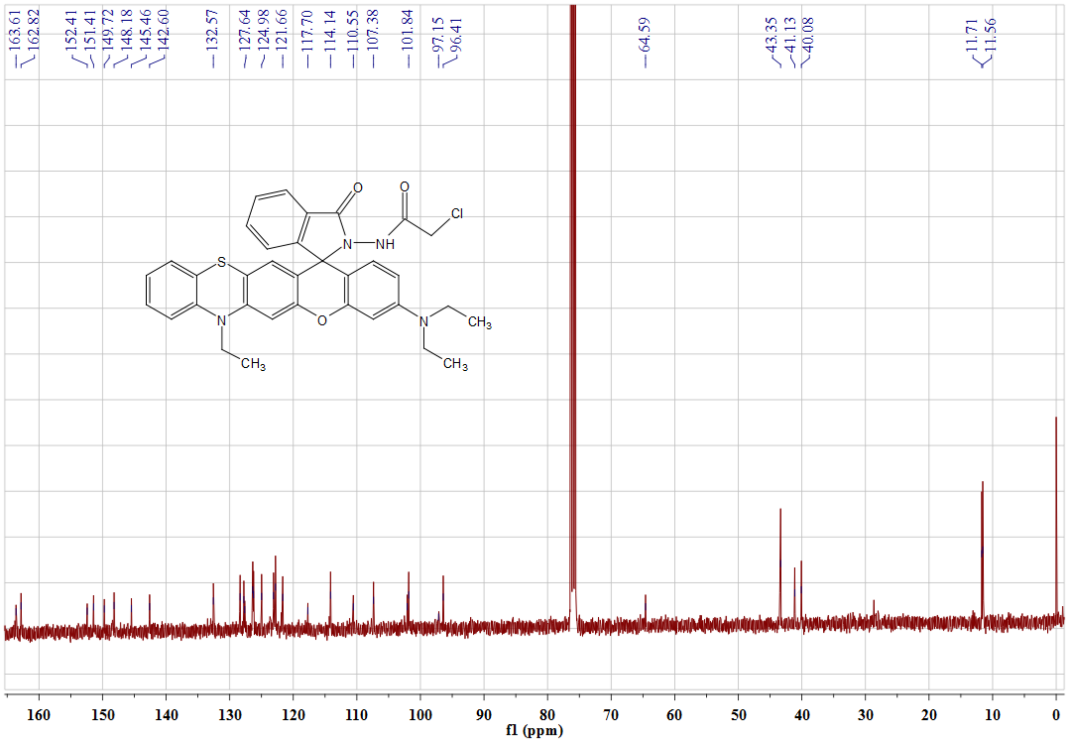

Fig. S5. ^{13}C NMR spectrum (100 MHz, $CDCl_3$) of compound **3**.

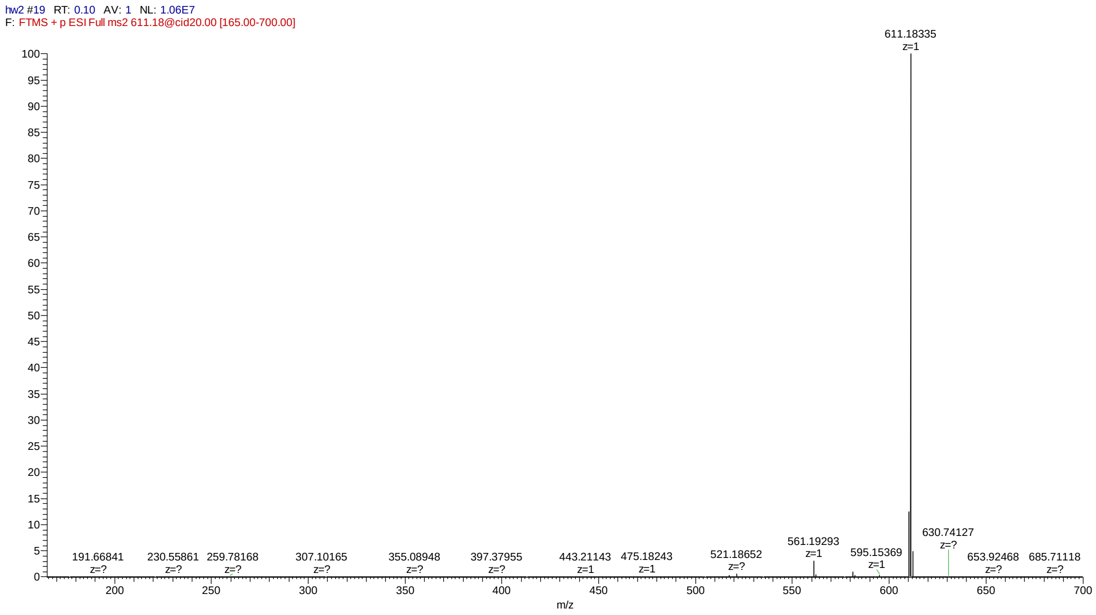

Fig. S6. HR-MS spectrum of compound **3**.

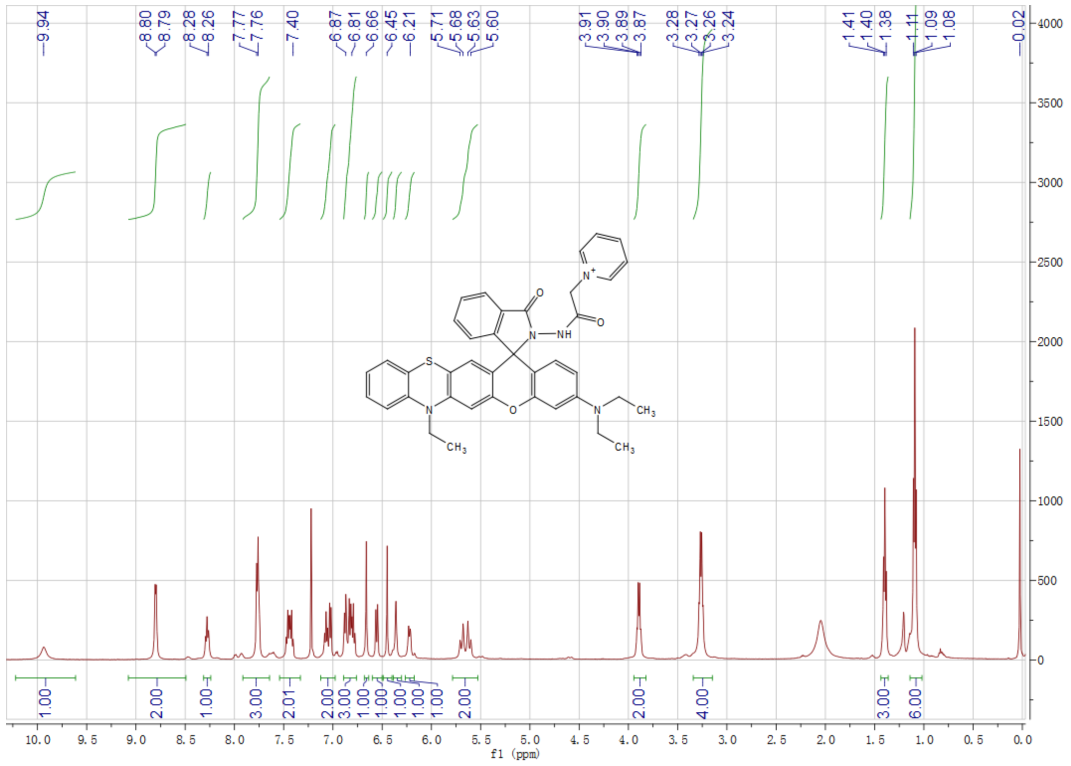

Fig. S7. ^{1}H NMR spectrum (400 MHz, $CDCl_3$) of **WD-HOCl**.

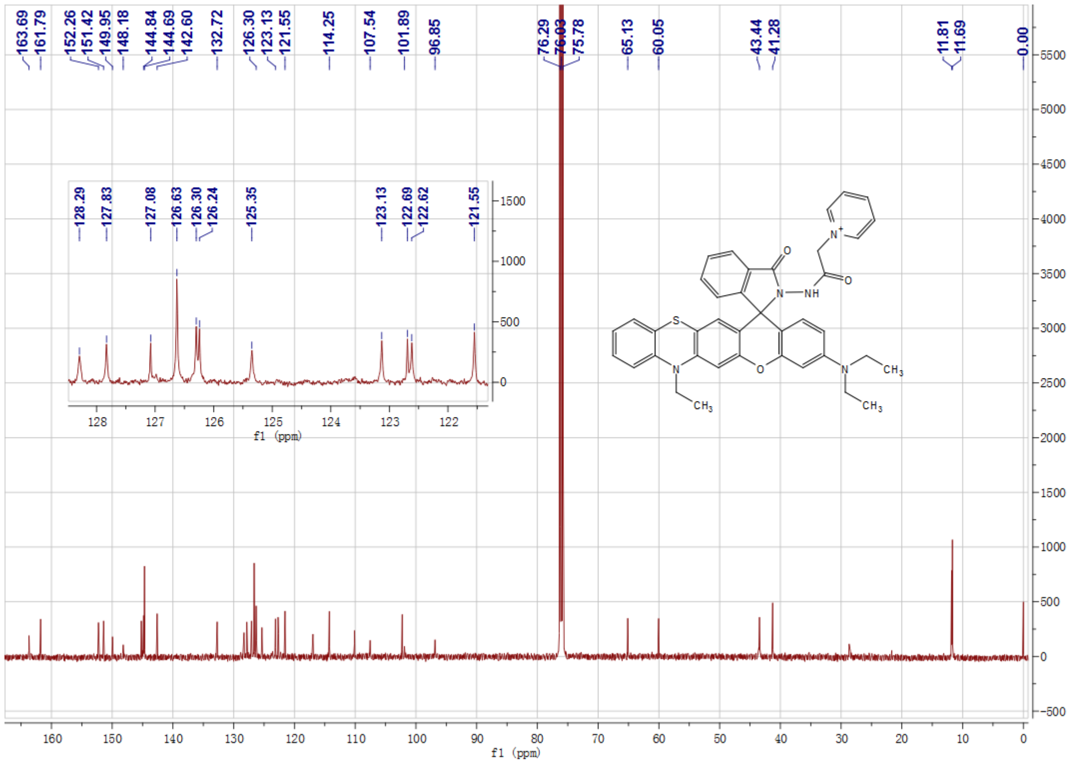

Fig. S8. ^{13}C NMR spectrum (100 MHz, $CDCl_3$) of **WD-HOCl**.

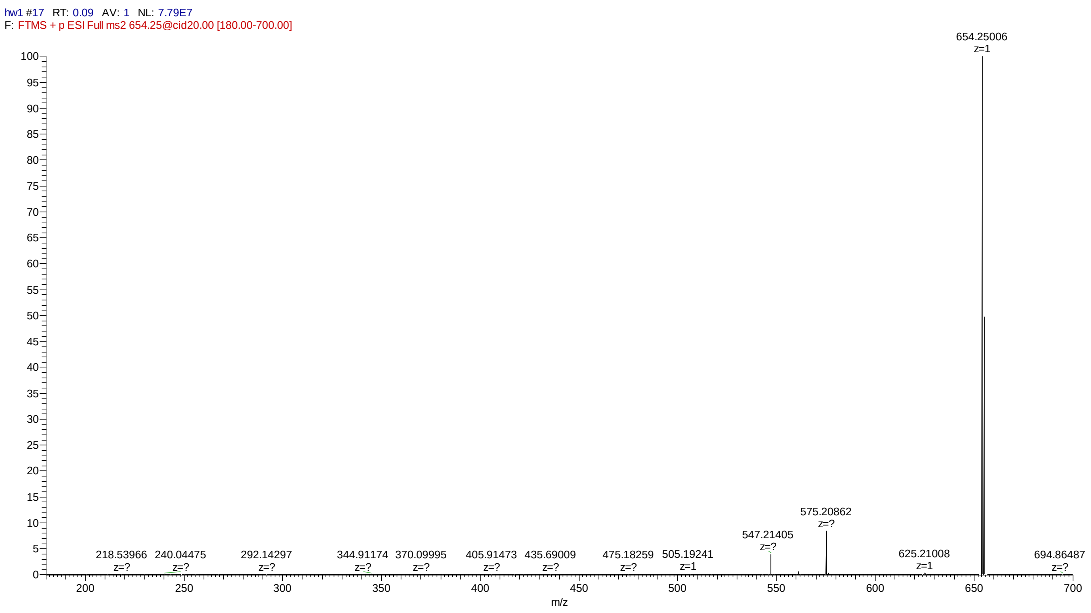

Fig. S9. HR-MS spectrum of **WD-HOCl**.

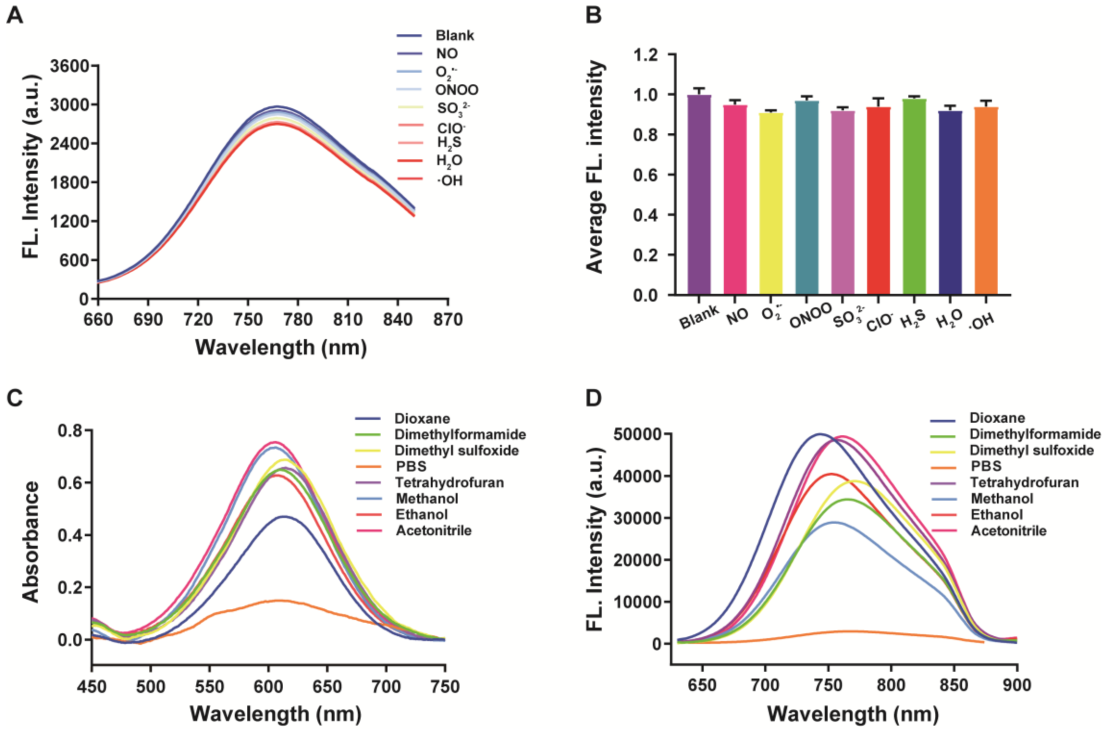

Fig. S10. Fluorescence intensities of **TJ730** (10 μM) in response to various agents in PBS (pH = 7.4, H_2O/CH_3CN = 2:1, v/v) (50 μM for $ONOO^-$; 100 μM for H_2O_2, $O_2^{\bullet-}$, H_2S, SO_3^{2-}, NO, HOCl, and •OH). (C) Absorbance spectra of **TJ730** (10 μM) in different solvents in the presence in the presence of TFA (1%); (D) Fluorescence emission spectra of **TJ730** (10 μM) in different solvents in the presence of TFA (1%). λ_{ex} = 606 nm, slit: 10 nm, 10 nm.

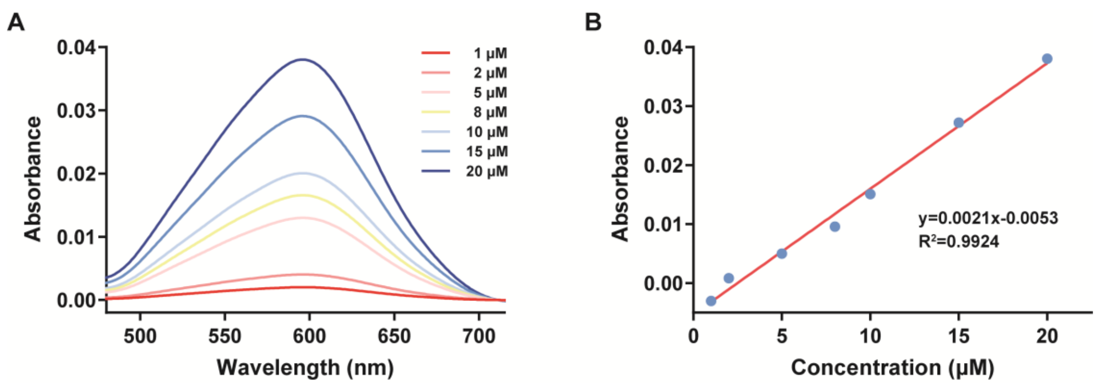

Fig. S11. (A) UV-vis spectra of **WD-HOCl** (1, 2, 5, 8, 10, 15, 20 μM) in the presence of HOCl (2 eq.) in PBS buffer solution; (B) Liner relationship of the absorbance at 606 nm with the concentrations of **WD-HOCl** (1, 2, 5, 8, 10, 15, 20 μM). λ_{ex} = 606 nm, slit: 10 nm, 10 nm.

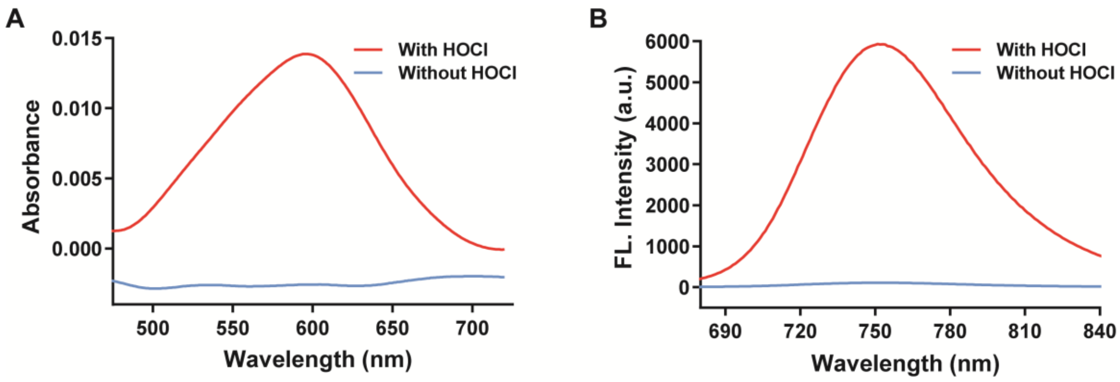

Fig. S12. (A) Absorption responses of probe **WD-HOCl** (10 μM) in the presence of HOCl (2 eq.) in PBS solution (10 mM, pH = 7.4); (B) Fluorescence spectra of probe **WD-HOCl** (10 μM) upon the addition of HOCl solution (2 eq.) in PBS buffer solution (10 mM, pH = 7.4) with H_2O/CH_3CN = 2:1 (v/v) as the co-solvent. λ_{ex} = 606 nm, slit: 10 nm, 10 nm.

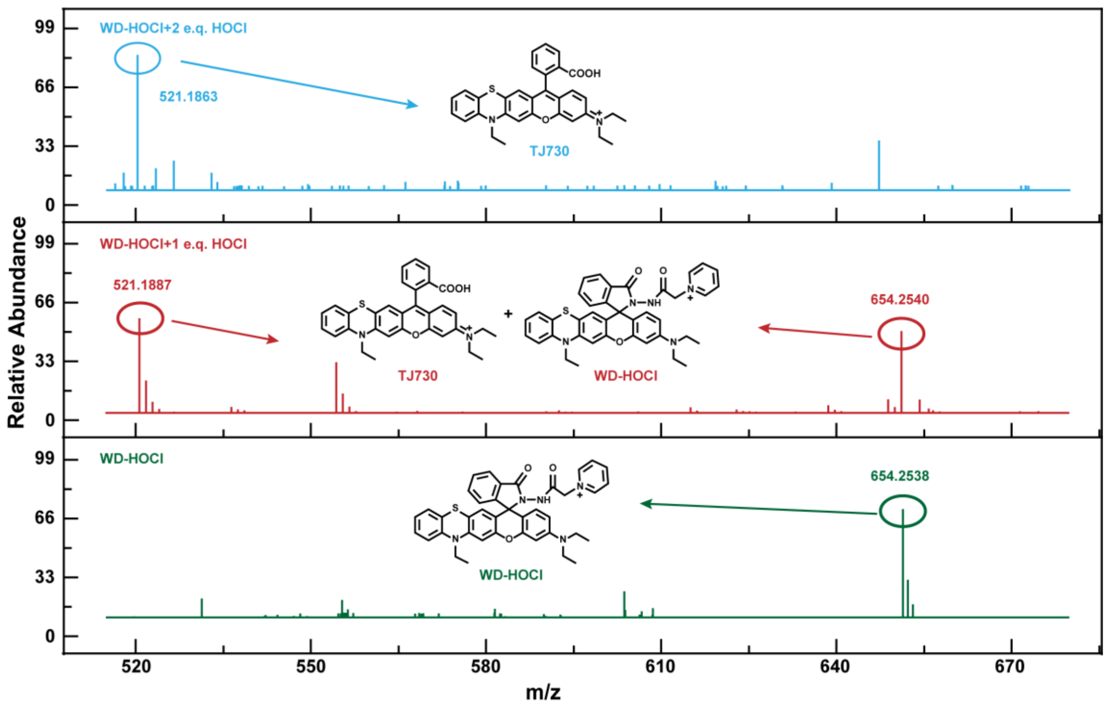

Fig. S13. HR-MS traces of **WD-HOCl** upon addition of top: HOCl (2 e.q.), middle: HOCl (1 e.q.) and bottom: HOCl (0 e.q.).

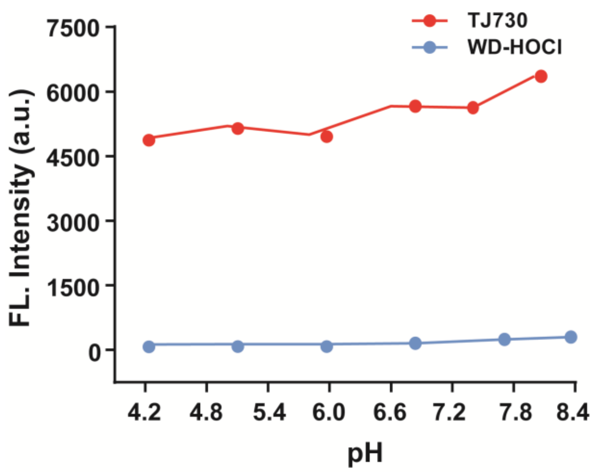

Fig. S14. Effects of pH on the fluorescence intensity of 10 μM **WD-HOCl** with (red line) or without HOCl (blue line). The excitation wavelength was 606 nm in 10 mM PBS (pH = 7.4, H_2O/CH_3CN = 2:1, v/v).

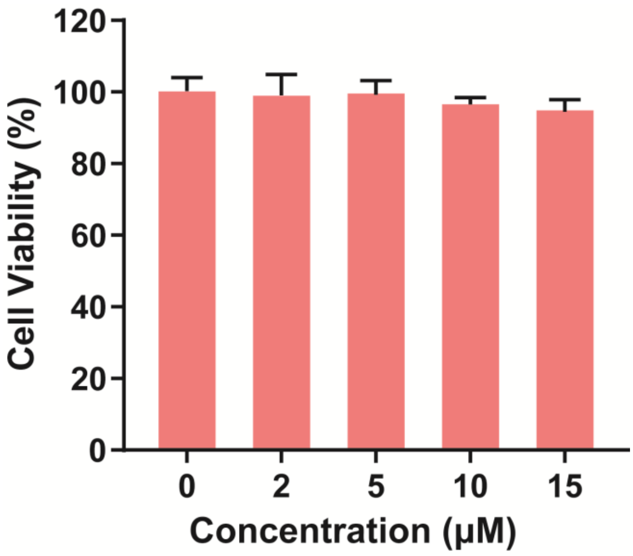

Fig. S15. MTT assay of BV-2 cells treated with different concentrations of **WD-HOCl** (0, 2, 5, 10, 15 μM).

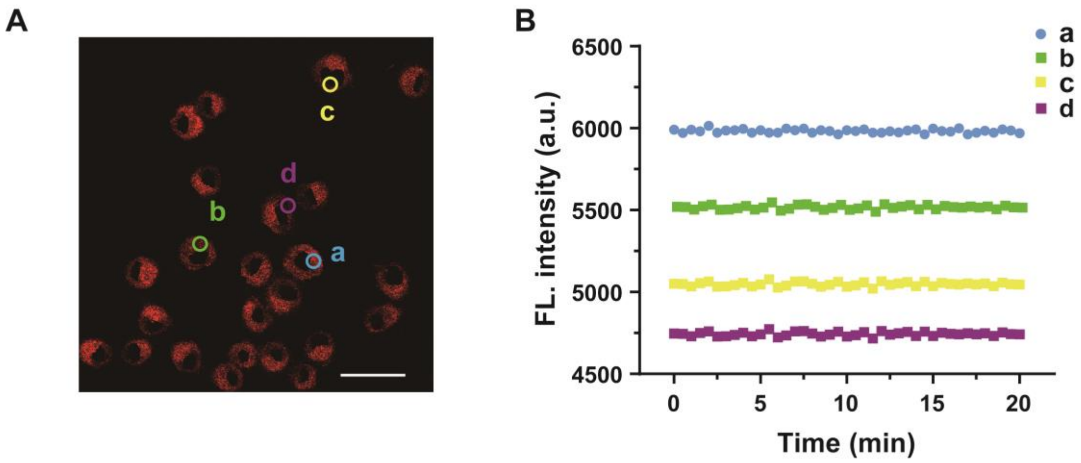

Fig. S16. (A) Confocal microscopic images of BV-2 cells labeled with 2 μg/mL PMA for 1 h and incubated with **WD-HOCl** for 30 min; (B) Fluorescence intensity from circle a, b, c andd as a function of time. The fluorescence intensity was collected with 30 sec intervals. Emissions were collected at the red channel (680–738 nm) with 606 nm excitation. Scale bar: 20 μm.

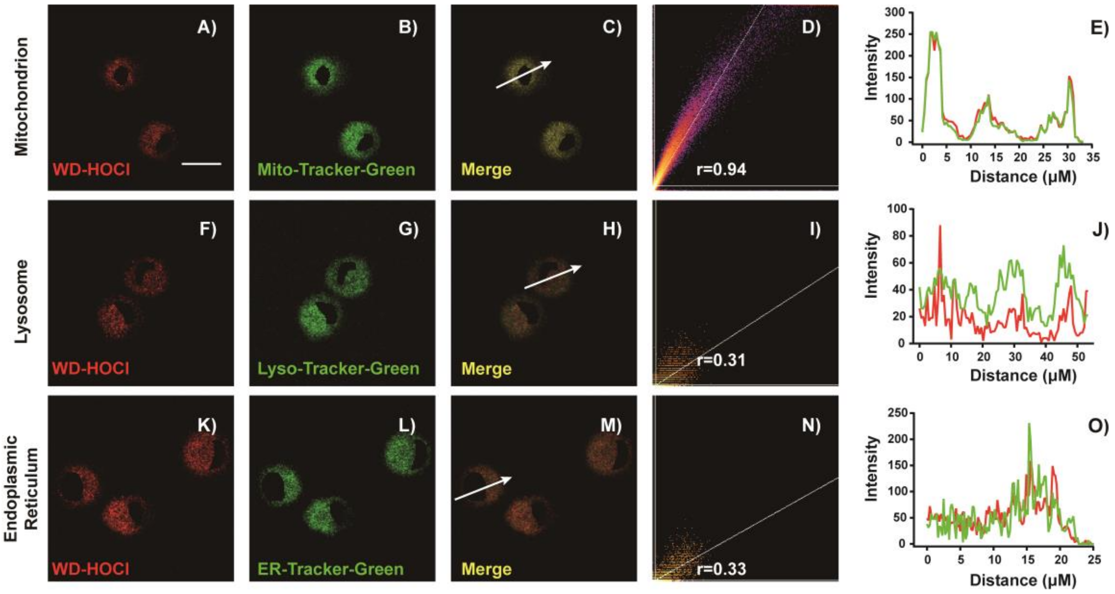

Fig. S17. Confocal microscopy images of co-localization BV-2 cell imaging of **WD-HOCl** and commercial dyes including (A-C) Mito-Tracker Green, (F-H) Lyso-Tracker Green, (K-M) ER-Tracker Green in BV2 cell. Green channel (505-600 nm, λ_{ex} = 504 nm) for Lyso-Tracker Green, Mito-Tracker Green and ER-Tracker Green; Red channel (680–738 nm, λ_{ex} = 606 nm). Scale bar: 50 μm. for **WD-HOCl**. (D, I, N) Fluorescence intensity correlation plot of **WD-HOCl** and commercial dyes. Scale bar, 50 μm. (E, J, O) Fluorescence intensity profile of the region of interest across cells in the red and green channels.

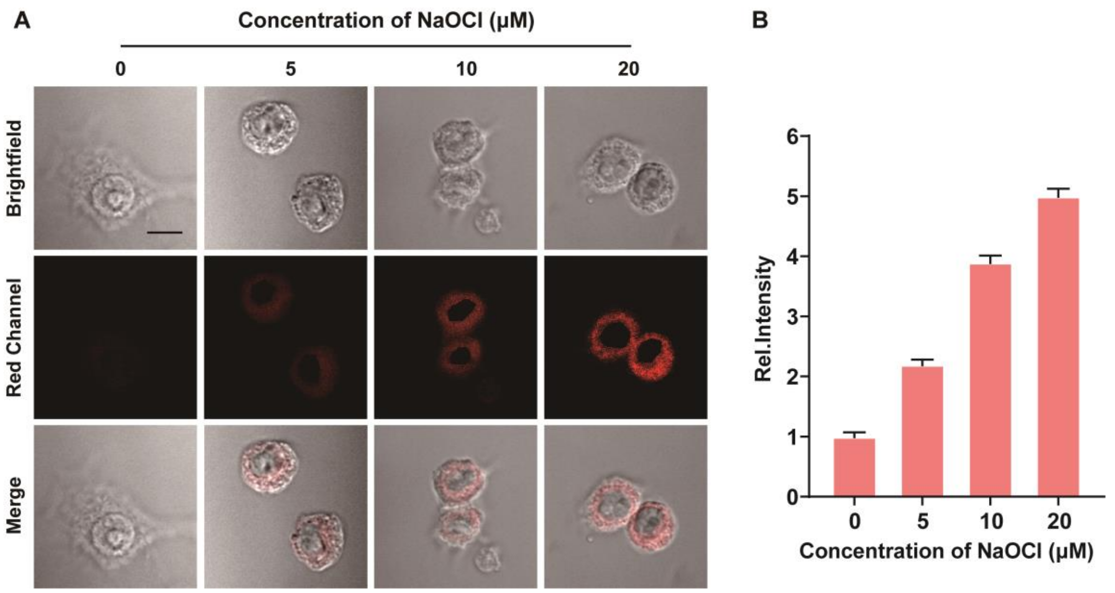

Fig. S18. Confocal microscopic images of exogenous HOCl detection in living BV-2 cells using **WD-HOCl**. (A) Cells were incubated with a) **WD-HOCl** (5 μM) alone for 30 min; b, c, d) **WD-HOCl** (5 μM) for 30 min and then NaOCl (5, 10, 20 μM) for another 30 min; (B) Histograms of average fluorescent intensities in (A). Emissions were collected in the red channel (680–738 nm) with 606 nm excitation. Scale bar: 50 μm.

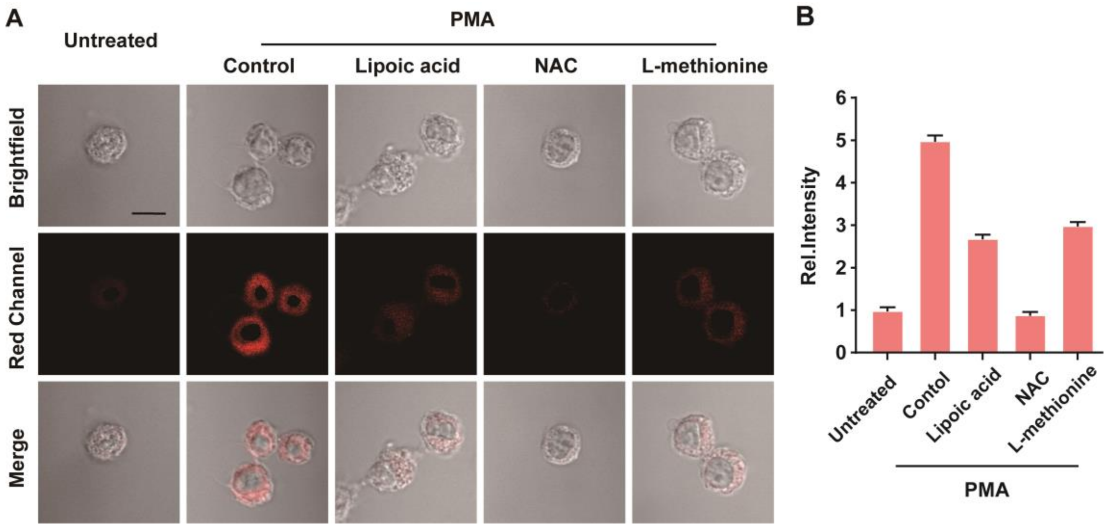

Fig. S19. (A) Confocal microscopic images of endogenous HOCl using **WD-HOCl** in BV-2 cells: (1) Untreated cells; (2) Treated with PMA (2 μg/mL) for 1 h; (3) the cells were treated with Lipoic acid (5 μg/mL) for 30 min and then with PMA (2 μg/mL) for 1 h. (4) the cells were treated with NAC (2 μg/mL) for 30 min and then with PMA (2 μg/mL) for 1 h. (5) the cells were treated with L-methionine (1 μg/mL) for 30 min and then with PMA (2 μg/mL) for 1 h. (B) Bars represent the relative fluorescence intensities between the corresponding cells and control cells. Emissions were collected in the red channel (680–738 nm) with 606 nm excitation. Scale bar: 50 μm.

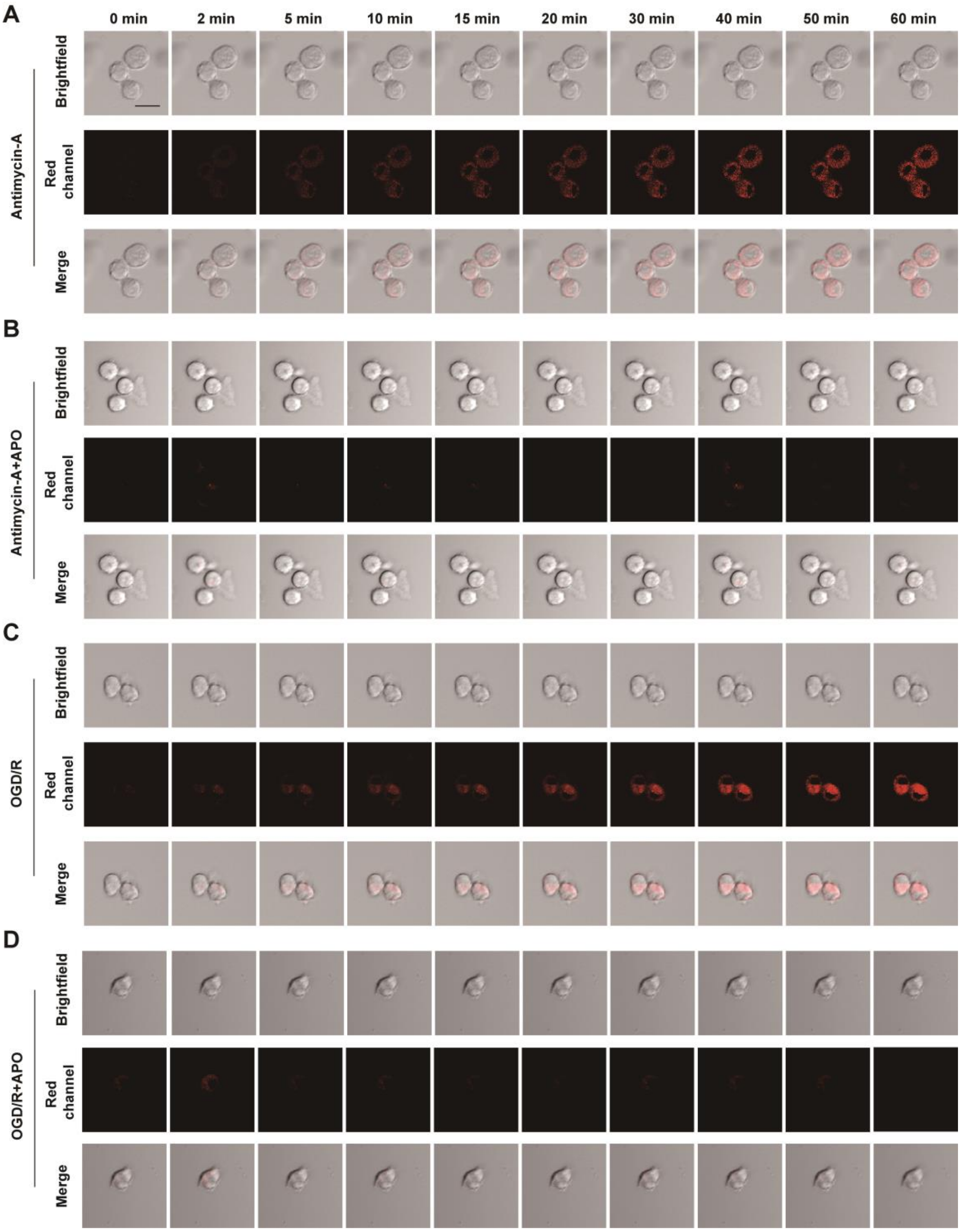

Fig. S20. Real-time imaging of BV-2 cells incubated with 10 μM **WD-HOCl** during (A) Antimycin A (5 μM); (B) Antimycin A+APO (10 mM); (C) OGD/R and (D) OGD/R+APO. Emissions were collected in the red channel (680–738 nm) with 606 nm excitation. For the fluorescent images, the experiment was repeated using three cultures; similar results were obtained each time. Scale bar: 20 μm. The error bars mean SD (n = 33 cells).

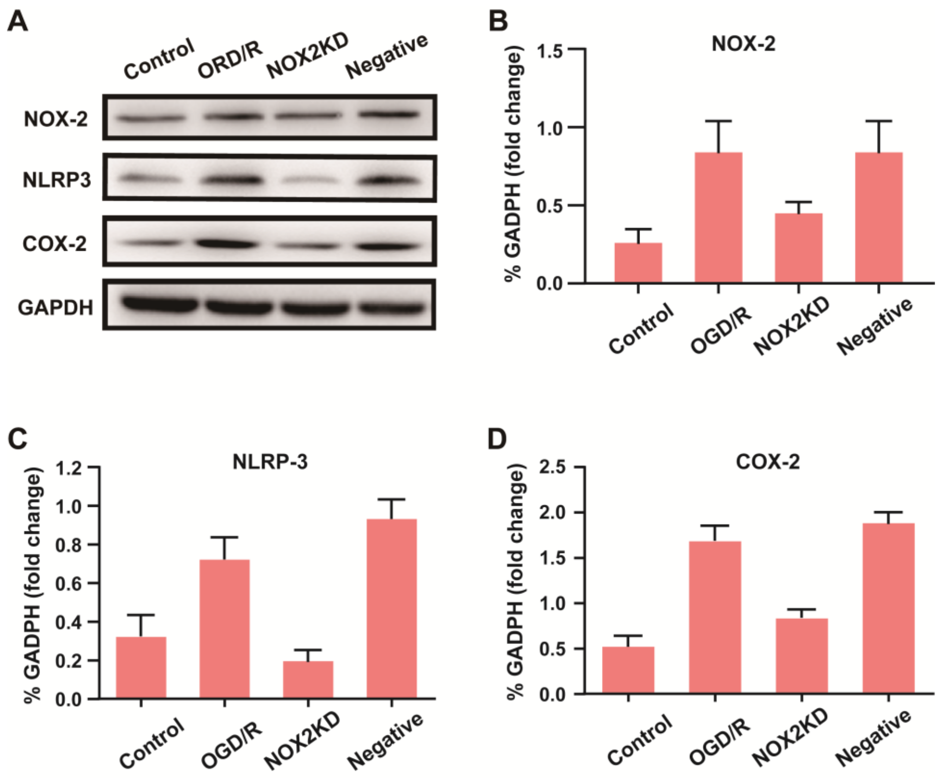

Fig. S21. (A) Western blotting illustrating the expression of NOX2, Beclin-1, NLRP3, and COX-2 in the BV-2 cells of (Figure 4B); GAPDH was used as a loading control; (B–D) Quantitative data of Western blot results for NOX-2 (B), NLRP-3 (C), and COX-2 (D) in the BV-2 cells. In B-D, error bars represent SD.

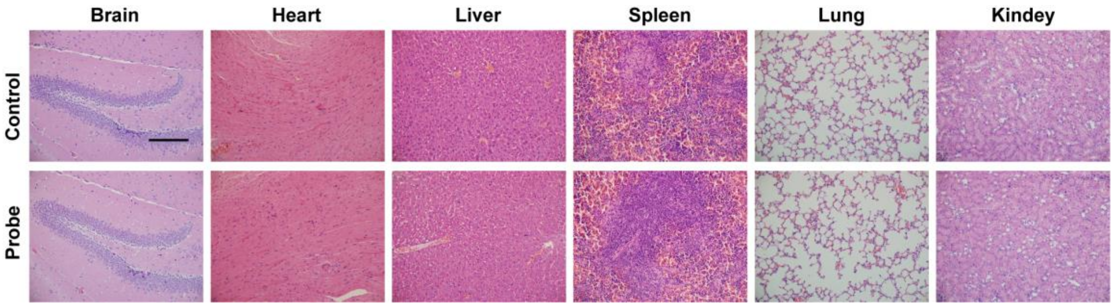

Fig. S22. H&E staining results of different organs collected from the control group and **WD-HOCl** (200 μL, 200 μM) treated group. Scale bar: 100 mm.

4. Supplementary Table

Table S1. Photophysical properties of the dye **TJ730** in different solvents with TFA (1%).

Solvent	λ_{abs}/nm	ε (M^{-1} cm^{-1})	λ_{em}/nm	Φ	Stokes Shifts (nm)
CH_3CN	606	75500	762	0.047	156
EtOH	606	62800	752	0.047	145
CH_3OH	606	73400	754	0.032	148
PBS	608	14900	767	0.002	158
DMSO	615	68700	769	0.032	155
DMF	610	65000	764	0.031	153
Dioxane	610	46900	745	0.046	135
THF	615	65600	758	0.045	143

5. References

(1) K. Raghavachari, *Theor Chem Acc.*, 2000, **103**, 361-363.

(2) J. Tomasi, B. Mennucci and R. Cammi, *Chem. Rev.*, 2005, **105**, 2999-3094.

(3) R. M. Uppu, *Anal. Biochem.*, 2006, **354**, 165-168.

(4) H. Maeda, K. Yamamoto, Y. Nomura, I. Kohno, L. Hafsi, N. Ueda, S. Yoshida, M. Fukuda, Y. Fukuyasu, Y. Yamauchi and N. Itoh, *J. Am. Chem. Soc.*, 2005, **127**, 68-69.

(5) J. C. Morris, *J. Phys. Chem.*, 1966, **70**, 3798-3805.

(6) J. Terao, A. Nagao, D.-K. Park and B. P. Lim, in Methods in Enzymology, Methods Enzymol., 1992, vol. **213**, pp. 454-460.

(7) J. M. Aubry, B. Cazin and F. Duprat, *J. Org. Chem.,* 1989, **54**, 726-728.

(8) R. Kohen, Y. Yamamoto, K. C. Cundy and B. N. Ames, *Proc. Natl. Acad. Sci. U. S. A.*, 1988, **85**, 3175-3179.

(9) B. Wang, T. Qiang, L. Ren, T. Liang, F. Cheng, M. Li and W. Hu, *Dyes Pigments*, 2022, **197**, 109957.

(10) Frisch, M. J., Trucks, G. W., Schlegel, H. B., Scuseria, G. E., Robb, M. A., Cheeseman, J. R. Scalmani, G., Barone, V., Mennucci, B., Petersson , G. A., et al. Gaussian 09, Revision A.1; Gaussian, Inc.: Wallingford, CT, 2009.

(11) S. K. Lee, W. J. Yang, J. J. Choi, C. H. Kim, S.-J. Jeon and B. R. Cho, *Org. Lett.*, 2005, **7**, 323-326.

(12) X. Xiong, L. Gu, Y. Wang, Y. Luo, H. Zhang, J. Lee, S. Krams, S. Zhu and H. Zhao, *J. Neuroinflammation*, 2016, **13**, 241.

(13) C. Li, W. Hu, J. Wang, X. Song, X. Xiong and Z. Liu, *Analyst*, 2020, **145**, 6125-6129.

(14) L. Gu, X. Xiong, H. Zhang, B. Xu, G. K. Steinberg and H. Zhao, *Stroke*, 2012, **43**, 1941-1946.

(15) X. Xiong, R. Xie, H. Zhang, L. Gu, W. Xie, M. Cheng, Z. Jian, K. Kovacina and H. Zhao, *Neurobiol. Dis.*, 2014, **66**, 43-52.

(16) C. M. Stary, L. Xu, L. Li, X. Sun, Y.-B. Ouyang, X. Xiong, J. Zhao and R. G. Giffard, *Cell. Neurosci.*, 2017, **82**, 118-125.

(17) R. Q. Han, Y. B. Ouyang, L. Xu, R. Agrawal, A. J. Patterson and R. G. Giffard, *Anesth. Analg.*, 2009, **108**, 280-287.

(18) W. Fang, X. Zhai, D. Han, X. Xiong, T. Wang, X. Zeng, S. He, R. Liu, M. Miyata, B. Xu and H. Zhao, *Theranostics*, 2018, **8**, 3530-3543.

(19) J. Terao, A. Nagao, D.-K. Park and B. P. Lim, in *Methods in Enzymology*, Academic Press, 1992, vol. **213**, pp. 454-460.

(20) R. Kohen, Y. Yamamoto, K. C. Cundy and B. N. Ames, *Proc. Natl. Acad. Sci. U. S. A.*, 1988, **85**, 3175-3179.

(21) C. Liu, X. Jiao, Q. Wang, K. Huang, S. He, L. Zhao and X. Zeng, *Chem. Commun.*, 2017, **53**, 10727-10730.
